# Interventions for Preventing Unintended, Rapid Repeat Pregnancy Among Adolescents: A Review of the Evidence and Lessons From High-Quality Evaluations

**DOI:** 10.9745/GHSP-D-17-00131

**Published:** 2017-12-28

**Authors:** Maureen Norton, Venkatraman Chandra-Mouli, Cate Lane

**Affiliations:** a United States Agency for International Development, Washington, DC, USA.; b World Health Organization, Geneva, Switzerland.; c Pathfinder International, Washington, DC, USA.

## Abstract

**Background:**

In 2017, of the 22.5 million parenting adolescents (ages 15–19) in 60 countries, approximately 4.1 million gave birth to a second or higher-order child. Adolescent pregnancy in general, and rapid repeat pregnancies specifically, expose young mothers and their children to multiple health and socioeconomic risks. The purpose of this article is to review the impact of interventions designed to prevent unintended, rapid repeat pregnancies among adolescents, including those aimed at changing norms to postpone “intended” closely spaced pregnancies to promote healthy spacing.

**Methods:**

We searched PubMed and other databases for evaluations of interventions published in English from 1990 through 2016. We included evaluations that assessed a programmatic intervention specifically designed to prevent rapid repeat pregnancy (occurring less than 24 months after the index birth) or birth (occurring less than 33 months after the index birth), or that reported on contraceptive continuation for at least 2 years. We first assessed the quality of the evaluations, then ranked the interventions based on the quality of the evaluation and the level of impact on repeat pregnancy or birth (statistically significant impact, positive trends but not statistically significant, or no impact) to identify the most effective interventions. Finally, we extracted program design and implementation lessons from the interventions included in the high-quality evaluations.

**Results:**

Our search identified 2,187 articles, of which 40 evaluations met the inclusion criteria (24=high quality, 14=moderate quality, 2=less rigorous). We found 14 high-quality evaluations in which the intervention achieved a statistically significant impact on repeat pregnancy or birth. These interventions fell into 5 broad categories: (1) contraceptive services and information, with proactive monitoring of contraceptive use and outreach to families; (2) postpartum contraceptive counseling and services provided soon after delivery; (3) activities that help adolescents improve planning skills, including preparing contraceptive plans; (4) social and behavioral change activities that help adolescents understand the role contraception can play in determining positive life outcomes, and the implications of their reproductive health decisions for their future; and (5) activities that provide mentoring, goal setting, and motivation.

**Conclusion:**

Effective interventions that prevent rapid adolescent childbearing link clinical contraceptive services with non-clinical activities that build planning skills, enhance understanding of the role that contraceptives can play in determining positive life outcomes, and provide mentoring and goal setting. Recognizing potentially synergistic effects, we recommend testing various combinations of these interventions, with access to contraception as the foundational activity.

## Abstract (precis)

Evidence shows that effective prevention of rapid repeat pregnancy among adolescents links adolescent-friendly clinical contraceptive services with non-clinical interventions that contribute to positive youth development.

## INTRODUCTION

The World Health Organization (WHO) and the United States Agency for International Development (USAID) define adolescents as those between the ages of 10 and 19 years of age.^1^ The Demographic and Health Surveys (DHS) gather birth data on only the 15–19-year-old subgroup. Focusing on this subgroup, a 2017 analysis of DHS data in 60 USAID-assisted countries in more- and less-developed regions found that 22.5 million adolescents ages 15–19 gave birth (Table 1). Another assessment of adolescent pregnancy in 42 low-resource countries estimated that 2.5 million even-younger adolescents, ages 12–15, also give birth annually.^2^

**TABLE 1.** Number and Percentage of Adolescents Ages 15–19 With a Birth in USAID-Assisted Countries, by Number of Births

Country	Total No. of Women 15–19	Number of Women 15-19 With:				Percentage of Women 15-19 With:			
		1 Birth	2 Births	3+ Births	Any Birth	1 Birth	2 Births	3+ Births	Any Birth
India 2005–2006	54,635,318	5,026,449	1,365,883	218,541	6,610,873	9.2	2.5	0.4	12.1
Bangladesh 2014	7,787,279	1,720,989	179,107	7,787	1,907,883	22.1	2.3	0.1	24.5
Nigeria 2013	9,955,173	1,353,904	298,655	49,776	1,702,335	13.6	3.0	0.5	17.1
Brazil 1996	8,510,147	966,753	221,264	34,041	1,222,057	11.4	2.6	0.4	14.4
DRC 2013–2014	4,718,045	778,477	188,722	33,026	1,000,226	16.5	4.0	0.7	21.2
Indonesia 2012	11,123,673	738,612	22,247	8,899	769,758	6.6	0.2	0.1	6.9
Tanzania 2015–2016	2,941,151	535,289	76,470	5,882	617,642	18.2	2.6	0.2	21.0
Pakistan 2012	10,722,312	493,226	85,778	10,722	589,727	4.6	0.8	0.1	5.5
Ethiopia 2016	5,805,546	516,694	63,861	5,806	586,360	8.9	1.1	0.1	10.1
Mozambique 2011	1,550,003	373,551	74,400	7,750	455,701	24.1	4.8	0.5	29.4
Angola 2015–2016	1,499,876	337,472	85,493	8,999	431,964	22.5	5.7	0.6	28.8
Uganda 2011	2,283,838	303,750	91,354	15,987	411,091	13.3	4.0	0.7	18.0
Philippines 2013	5,138,070	349,389	41,105	5,138	395,631	6.8	0.8	0.1	7.7
Kenya 2014	2,488,748	301,139	57,241	4,977	363,357	12.1	2.3	0.2	14.6
Madagascar 2008–2009	1,332,247	266,449	62,616	17,319	346,384	20.0	4.7	1.3	26.0
Niger 2012	1,043,873	246,145	82,466	13,362	341,973	23.6	7.9	1.3	32.8
Mali 2012-2013	971,050	241,791	67,974	11,653	321,418	24.9	7.0	1.2	33.1
South Africa 1998	2,372,020	298,875	9,488	2,372	310,735	12.6	0.4	0.1	13.1
Côte d'Ivoire 2011–2012	1,328,997	239,751	62,330	4,386	306,467	18.0	4.7	0.3	23.1
Egypt 2014	4,369,133	253,410	39,322	–	292,732	5.8	0.9	0.0	6.7
Colombia 2015	1,983,614	228,116	37,689	4,166	269,970	11.5	1.9	0.2	13.6
Malawi 2015–2016	1,091,464	223,750	16,372	2,183	242,305	20.5	1.5	0.2	22.2
Burkina Faso 2010	1,087,568	174,663	26,645	2,175	203,484	16.1	2.5	0.2	18.7
Nepal 2016	1,579,950	170,635	26,859	4,740	202,234	10.8	1.7	0.3	12.8
Zambia 2013–2014	859,608	176,220	21,490	2,579	200,289	20.5	2.5	0.3	23.3
Turkey 2003	3,230,340	155,056	19,382	9,691	184,129	4.8	0.6	0.3	5.7
Guinea 2012	654,601	142,572	36,789	3,731	183,092	21.8	5.6	0.6	28.0
Afghanistan 2015	1,992,100	123,510	31,874	3,984	159,368	6.2	1.6	0.2	8.0
Ghana 2014	1,377,890	139,167	15,157	1,378	155,702	10.1	1.1	0.1	11.3
Peru 2012	1,366,132	129,783	17,486	410	147,679	9.5	1.3	0.0	10.8
Guatemala 2014–2015	841,331	116,945	15,985	3,365	136,296	13.9	1.9	0.4	16.2
Zimbabwe 2015	773,876	119,177	10,834	–	130,011	15.4	1.4	0.0	16.8
Yemen 2013	1,567,150	97,163	26,642	4,701	128,506	6.2	1.7	0.3	8.2
Senegal 2016	796,791	81,273	15,936	1,594	98,802	10.2	2.0	0.2	12.4
Honduras 2011–2012	482,308	79,870	11,527	482	91,880	16.6	2.4	0.1	19.1
Dominican Rep. 2013	489,499	70,977	10,133	489	81,599	14.5	2.1	0.1	16.7
Benin 2011–2012	602,731	66,059	12,235	1,326	79,621	11.0	2.0	0.2	13.2
Bolivia 2008	552,238	65,109	12,591	1,491	79,191	11.8	2.3	0.3	14.3
Uzbekistan 1996	1,234,969	68,170	6,175	2,470	76,815	5.5	0.5	0.2	6.2
Liberia 2013	257,899	57,511	8,769	258	66,538	22.3	3.4	0.1	25.8
Haiti 2012	577,907	56,635	7,513	1,156	65,303	9.8	1.3	0.2	11.3
Morocco 2003–2004	1,451,561	56,611	5,806	1,306	63,724	3.9	0.4	0.1	4.4
Nicaragua 2001	305,869	50,774	10,828	1,499	63,101	16.6	3.5	0.5	20.6
Togo 2013-2014	403,711	48,163	5,410	–	53,572	11.9	1.3	0.0	13.3
Cambodia 2014	722,097	48,597	4,333	72	53,002	6.7	0.6	0.0	7.3
Burundi 2010	597,098	35,348	4,419	–	39,767	5.9	0.7	0.0	6.7
Eritrea 2002	311,661	28,673	5,298	623	34,594	9.2	1.7	0.2	11.1
Rwanda 2014–2015	631,072	32,816	1,262	–	34,078	5.2	0.2	0.0	5.4
Mauritania 2000–2001	200,101	18,609	6,603	1,001	26,213	9.3	3.3	0.5	13.1
Kazakhstan 1999	576,648	23,643	1,499	–	25,142	4.1	0.3	0.0	4.4
Ukraine 2007	930,583	20,938	3,629	–	24,567	2.3	0.4	0.0	2.6
Jordan 2012	500,920	14,527	3,006	–	17,532	2.9	0.6	0.0	3.5
Tajikistan 2012	398,873	14,479	878	120	15,476	3.6	0.2	0.0	3.9
Swaziland 2006–2007	82,377	13,180	2,059	–	15,240	16.0	2.5	0.0	18.5
Azerbaijan 2006	305,795	9,357	2,110	275	11,743	3.1	0.7	0.1	3.8
Kyrgyzstan 2012	228,641	8,917	183	91	9,191	3.9	0.1	0.0	4.0
Moldova 2005	93,573	4,323	159	–	4,482	4.6	0.2	0.0	4.8
Timor-Leste 2009–2010	71,039	3,104	717	199	4,021	4.4	1.0	0.3	5.7
Albania 2008–2009	117,252	2,075	94	–	2,169	1.8	0.1	0.0	1.9
Armenia 2015–2016	83,885	2,097	–	–	2,097	2.5	0.0	0.0	2.5
**Total**	**171,989,221**	**18,320,709**	**3,622,151**	**523,979**	**22,466,839**				**13.1**
**Total no. (%) with subsequent births**				**4,146,130 (2%)**					

Abbreviations: DRC, Democratic Republic of the Congo; USAID, United States Agency for International Development.

Sources of data: Population of women ages 15–19 from 2017 U.S. Census Bureau data; number of women ages 15–19 with births from the most recent Demographic and Health Survey for each country. Analysis conducted by the USAID Knowledge Management Services II project.

Not all adolescent births are first births. A significant number of adolescents, having begun early childbearing, are at risk of experiencing a rapid repeat pregnancy. In fact, in 2017, of the 22.5 million total adolescent pregnancies occurring in 60 USAID-assisted countries, approximately 4.1 million adolescents gave birth to a second or higher-order child (Table 1). While the *percentages* of adolescents at the country level who have second or higher-order births are relatively small (for example, ranging from 0.1% in Albania and Kyrgyzstan to 9.2% in Niger), the *numbers* of adolescents experiencing a subsequent birth can be large (reaching nearly 1.6 million in India). While many adolescent births occur within marriage where sexual activity and pregnancy are socially sanctioned, it is likely that many of the closely spaced pregnancies are unintended. An analysis of 27 DHS surveys assessed the proportion of women ages 15–49 with unmet need for contraception who were within 1 year of their last delivery and those intending to use a contraceptive method within the next 12 months. The analysis found that “only trivial proportions of both of these groups want another birth within two years.”^3^

In 2017, 22.5 million adolescents ages 15–19 in 60 countries gave birth and, of these, approximately 4.1 million gave birth to a second or higher-order child.

Adolescent pregnancy exposes young mothers and their children to multiple health and socioeconomic risks. In the most recent and largest analysis known to date (>124,000 mothers in 29 countries), conducted by WHO, adolescent mothers ages 10–19 years had higher risks than mothers ages 20–24 years of eclampsia, puerperal endometritis, systemic infections, low birthweight, preterm delivery, and severe neonatal conditions.^4^ In addition, adolescent mothers are less likely to complete school or participate in the labor force, and earn less in their jobs when they do work.^5^^,^^6^ Short inter-pregnancy intervals, or rapid repeat pregnancies, also pose their own set of risks including increased risks of preterm birth, low birthweight, small for gestational age, and infant and early childhood mortality.^7^^–^^12^

**FIGURE 1:**
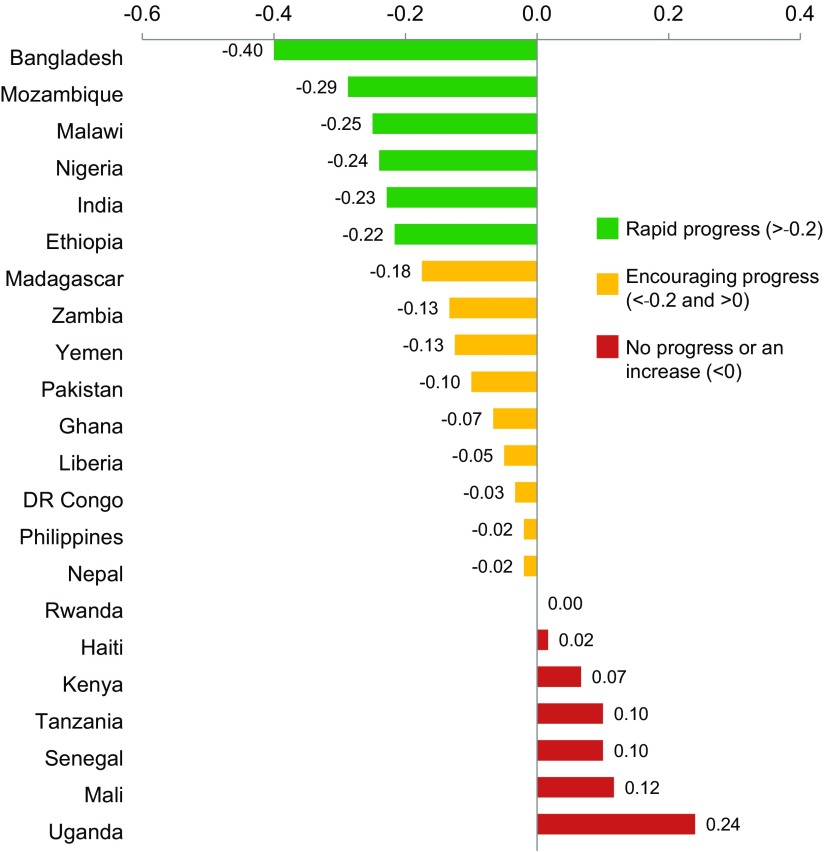
Annual Percentage Point Change in Adolescent Repeat Pregnancy Among USAID Priority Countries,^a^ 2013–2016 ^a^ Data are shown for 22 of 24 USAID priority countries; no data were available for South Sudan and trend data were unavailable for Afghanistan. Source of data: Trends are extrapolated from the last 2 survey data points from Demographic and Health Surveys and Reproductive Health Surveys. Analysis conducted by the USAID Knowledge Management Services II project.

In the United States, the Healthy People 2020 initiative has set forth, for all women and adolescents, a national goal of reducing the proportion of pregnancies conceived within 18 months of a previous birth (27 months between births) by 10%, from 33.1% of all births between 2006 and 2010 to 29.8% of all births in 2020.^13^ In low-resource settings, however, few programmatic or policy activities have been devoted to helping pregnant and parenting adolescents (married or unmarried) make an informed choice about contraceptive use to delay or space subsequent pregnancies. Between 2013 and 2016, in 22 USAID priority countries, the percentages of adolescents ages 15–19 with birth intervals less than 24 months decreased notably (by at least 0.2 percentage points) in only 6 countries. Nine other countries were making encouraging progress while 7 other countries witnessed no progress or an increase (Figure 1).

The purpose of this article is to review interventions that were designed to prevent rapid repeat pregnancies among adolescents. This includes interventions focused on educating adolescents, families, and communities about the risks of closely spaced pregnancies and changing norms to promote healthy pregnancy spacing. Our goal is to make available to program designers and managers practical, evidence-based programmatic lessons to help adolescents and young adults avoid rapid childbearing.

## METHODS

### Study Objectives

Our review addressed the following questions:

- In high-quality evaluations, what is the impact of programmatic interventions on prevention of rapid repeat pregnancy among adolescents?
- In high-quality evaluations, which programmatic interventions are the most effective, and which are less effective, in preventing rapid repeat pregnancy among adolescents?
- What lessons can we learn?

### Search Strategy

We conducted computerized searches of PubMed, PsycINFO, Sociological Abstracts, CINAHL, and Cochrane Reviews to identify evaluations of interventions that were published in English from 1990 to December 2016. Search terms included: “birth-to-birth interval,” “birth-to-pregnancy interval,” “birth interval,” “short birth interval,” “rapid, repeat pregnancy,” “repeat pregnancy,” and “adolescent repeat pregnancy.” All retrieved literature was screened at the abstract level for relevance, and articles that recorded only shifts in knowledge were excluded. After this initial screening, we assessed eligibility of the remaining articles by reviewing the abstracts a second time to identify those articles that met the inclusion criteria for this review.

### Inclusion/Exclusion Criteria

Using the following criteria, we included evaluations that:

- Evaluated a programmatic intervention specifically designed to prevent rapid repeat pregnancy or birth, or that reported on contraceptive continuation for 2 years or more
- Were published in a peer-reviewed journal between 1990 and 2016
- Were conducted in high-, middle-, or low-income countries
- Presented quantitative data that measured:
  - Subsequent pregnancies after the index birth at 6, 9, 12, 18, 24, or 30 months or prior to 6 months for Lactational Amenorrhea Method (LAM) interventions, or
  - Births at 18 months or more after the index birth, or
  - Contraceptive use at 6, 9, 12, 18, 24, or 30 months postpartum

If we encountered several evaluations of the same program, we included the most recent evaluation.

We excluded evaluations if they:

- Did not assess interventions that were explicitly designed to prevent adolescent or adult rapid repeat pregnancy or short birth or pregnancy intervals, or did not report on contraceptive continuation for at least 2 years
- Did not measure:
  - Pregnancy at 6, 9, 12, 18, 24, or 30 months after the index birth, or prior to 6 months for LAM interventions, or
  - Births at 18 months or more after the index birth, or
  - Continued use of contraception for at least 2 years
- Were implemented with incarcerated populations or populations in drug or alcohol treatment programs
- Evaluated a single contraceptive method and its effects on repeat pregnancy, and did not describe accompanying service delivery interventions
- Were designed to prevent the first adolescent pregnancy
- Were designed to increase postpartum family planning use by providing a range of contraceptives but did not describe educational or other programmatic interventions specifically aimed at preventing rapid repeat pregnancy

It was beyond the scope of this study to consider broad literature reviews on adolescent pregnancy and related topics. Also, while some evaluations reported pregnancy termination data, most did not do so, so it was not possible to assess how these events influenced program outcomes.

### Definitions

**Rapid repeat pregnancy or birth:** Pregnancy occurring less than 24 months after a live birth, or birth occurring less than 33 months after a live birth. (These are equivalent measures, translating into almost 3 years between births.) In 2005, a WHO technical consultation reviewed evidence on birth spacing and health outcomes and concluded^14^:

After a live birth, the recommended interval before attempting a pregnancy is at least 24 months … to reduce the risk of adverse maternal, perinatal, and infant outcomes.

Many of the evaluations in this review measured pregnancy occurring at 24 months after the index birth. Some measured births occurring during a specified time after the index birth. While DHS collects birth-to birth-data, other researchers often gather birth-to-pregnancy data. In this article, we will discuss both, depending on the categorization used in the evaluation.

**Intervention:** An activity, or set of activities, intended to achieve a defined outcome; in this case, the desired outcome is the reduction or prevention of rapid repeat pregnancy or birth in a specified population. Often, multiple, individual interventions are implemented as part of a broader intervention. For example, a postpartum contraceptive intervention might include multiple interventions such as counseling, contraceptive services, education of partners and families, and preparing a contraceptive plan. For ease of discussion, we define all of these activities as interventions and point out when they are implemented as part of a broader programmatic intervention.

**Evaluation:** The assessments of interventions included in this review.

### Data Collection and Analysis

We undertook a quality review of the evaluations included in this review using various study quality assessment tools as guides, such as those from the U.S. National Institutes of Health.^15^ Specifically, we rated the quality of each evaluation against the following 6 criteria:

1. Use of quantitative analyses to attribute change to the intervention (yes/no)
2. Randomization of individual subjects (yes/no)
3. Use of concurrent comparison group (yes/no)
4. Sample size ≥99 (yes/no)
5. Baseline and endline evaluation (yes/no)
6. Length of subject observation; measurement of:
  - Repeat pregnancy not <9 months after the index birth (<6 months for LAM evaluations) (yes/no)
  - Birth not <24 months after the index birth (yes/no)
  - 2-year continued use of contraceptives (yes/no)
  - Birth or pregnancy during not <3 years of program implementation (yes/no)

Evaluations with 5–6 “yes” classifications with respect to the criteria were rated high quality; 3–4 “yes” classifications were rated moderate quality; and 1–2 “yes” classifications were rated less rigorous.

We then extracted information on the intervention approaches implemented in the included evaluations as well as data on the impact of the interventions on repeat pregnancy or birth. We ranked the evaluations by their quality and by impact of the intervention on repeat pregnancy or birth (statistically significant impact, positive but not statistically significant trends, or no impact). In a separate analysis, we examined the magnitude of effect of interventions assessed in high-quality evaluations that measured similar outcomes at similar time periods—that is, they measured repeat pregnancy or birth at 18–24 months postpartum.

After ranking the quality of the evaluations and categorizing the level of impact, we then focused only on the high-quality evaluations. We classified interventions that achieved a statistically significant impact on repeat pregnancy, birth, or 2-year or more contraceptive continuation rates as “most effective” for preventing rapid repeat pregnancy. In contrast, we classified those interventions (assessed in high-quality evaluations) that showed either no impact or only positive trends toward preventing repeat pregnancy/birth but that did not achieve statistical significance as “less effective.”

To draw program design and implementation lessons from the interventions that could be applied in future programs, we examined the various types of interventions included in the high-quality evaluations. If we found 3 or more high quality evaluations of similar types of interventions that addressed similar design or implementation issues, we considered such findings to convey a lesson. For each lesson, we described the interventions and their impact in greater detail. As relevant, we added findings from high-quality evaluations of interventions that were less effective to illustrate how certain elements that were lacking could reduce effectiveness of the intervention. For some lessons, we identified evaluations that provided evidence but did not discuss the evaluations in detail. Finally, we identified additional factors, discussed in the high-quality evaluations, that may have reduced effectiveness of the interventions.

## RESULTS

### Selection and Characteristics of the Evaluations

**FIGURE 2:**
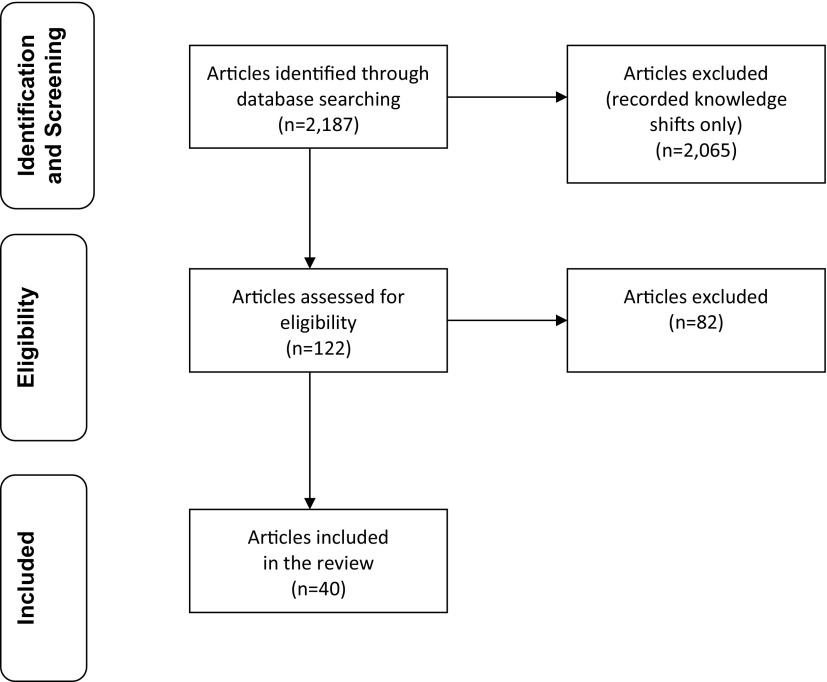
Article Selection Process

Our database search identified a total of 2,187 articles (Figure 2). After excluding evaluations that recorded only shifts in knowledge, 122 articles remained, of which 40 met the inclusion criteria. In addition, we drew separately on 2 analyses^16^^,^^17^ of interventions that were included in our review of evaluations but were published as separate studies. These analyses, which we refer to as “studies,” advance understanding of the factors that contributed to impact.

Of the 40 evaluations included in this review, 15 were randomized controlled trials,^18^^–^^32^ 15 used quasi-experimental designs that included comparison arms,^33^^–^^47^ 9 used pre-post or other designs,^48^^–^^56^ and 1 used longitudinal survey data to measure contraceptive continuation for 2 years.^57^ Based on our quality review of the 40 evaluations, we rated 24 evaluations as high quality^18^^–^^33^^,^^36^^–^^40^^,^^43^^,^^44^^,^^57^ (15 of which were randomized controlled trials), 14 as moderate quality,^34^^,^^35^^,^^41^^,^^42^^,^^45^^–^^49^^,^^51^^–^^54^^,^^56^ and 2 as less rigorous.^50^^,^^55^ (See Supplement Table 1 for detailed results of our quality assessment.) The outcome measured by most evaluations consisted of percentages or numbers of subjects in the intervention and comparison groups experiencing a repeat birth or pregnancy at a specified time period, usually 24 months, after the index birth. See Table 2 for an overview of intervention approaches employed in the 40 evaluations included in this review.

**TABLE 2.** Intervention Approaches Used in the Evaluations Reviewed (N=40)

Interventions	Description
**Comprehensive Services**	
Provision of multiple services	May include contraceptive services, contraceptive education, maternal/infant/child health services, child care, social work services, and/or home visitation
**Contraceptive Information and Services**	
Provision of contraceptive services	Through clinical or home-based delivery, includes counseling on correct method use and side effects
Comprehensive sexuality education	Includes contraceptive education, availability and correct use of contraceptives, sexual health and responsibility, dispelling myths about contraceptives
Pregnancy testing	Provision of monthly pregnancy tests
Surveys of contraceptive use	Regular assessments to monitor contraceptive use
Counseling on use of LAM with or without emergency contraception	Contraceptive services organized to provide LAM counseling and education; may include take-home supply of emergency contraception
Postpartum contraception	Provision of contraceptive services and counseling in the immediate or extended(24 months) postpartum period
**Planning for Contraceptive Use and Pregnancy Planning**	
Antenatal contraceptive plan	In antenatal period, clients encouraged to articulate fertility intentions and prepare contraceptive plan to achieve fertility intentions
“Implementation Intention Formation” training	Training in “if-then” planning: “If I am brushing my teeth in the morning, then I will take my contraceptive pill.”^21^
Planning the next pregnancy	Clients encouraged to state the preferred timing of their next pregnancy
**Community-Based Social and Behavioral Change Communication**	
Interpersonal counseling on fertility return after live birth	Clients advised that fertility can return before menses returns and, to avoid unintended pregnancy, not to wait for menses return before starting use of contraceptives
Interpersonal counseling on healthy pregnancy spacing	Clients advised of health/quality of life benefits of spacing next pregnancy 24 months after last birth, and potential adverse outcomes for mother and infant of closely spaced births
Social networks/group discussions in homes of village influentials	Group discussions to convey accurate information about contraceptive methods, advance understanding of the positive benefits of contraceptive use, and encourage discussions about contraceptive use with husbands and friends
Peer counseling interactions	Counseling by and discussion with social groups who have similar age, background, and social status as subjects
**Motivating, Mentoring, Goal Setting**	
Cell phone counseling	Using cell phones, project counselors use standardized curricula (based on teen's goals and needs) to hold weekly counseling calls for the first 6 months, followed by calls every 2 weeks for the next 12 months, for a total of 42 counseling sessions over 18 months. Cell phone service provided 450 minutes per month of use without surcharge.
Goal setting	Nurses/social workers assist teens in preparing short- and long-term plans to achieve life goals
Mentorship curriculum	Use of planned mentorship curriculum by providers who have had similar life experiences and often serve as “big sisters”
Home visitation	Periodic visits by nurses/community health workers to the homes of postpartum women, usually once a month over a 1–2-year period, to provide education, counseling, and/or contraceptive services
Motivational interviewing	Use of a counseling style that “emphasizes an individual's personal goals and self-efficacy in relation to complex behaviors”^20^
Skills training and job placement	Educational support for adolescent mothers under age 16 to return them to school, and skills training and job placement for adolescent mothers over age 18

Abbreviation: LAM, Lactational Amenorrhea Method.

### Impact of Interventions on Prevention of Rapid Repeat Pregnancy Among Adolescents

We found 14 high-quality evaluations in which the intervention achieved a statistically significant impact on rapid repeat pregnancy or birth up to 24 months after the index birth, or on contraceptive continuation for 24 months (Supplement Table 2). Eight of these were randomized controlled trials,^18^^–^^25^ 2 were cohort studies,^40^^,^^57^ and 4 used a quasi-experimental design.^33^^,^^36^^,^^37^^,^^39^ Of these 14 high-quality evaluations of interventions that achieved impact, 5 were conducted in developing-country settings (Bangladesh,^39^^,^^57^ Egypt,^22^ India,^37^ and Jamaica^40^); 8 were conducted in the United States^18^^–^^20^^,^^23^^–^^25^^,^^33^^,^^36^; and 1 was conducted in the United Kingdom.^21^ Nine evaluations focused exclusively on adolescents^18^^–^^21^^,^^23^^,^^24^^,^^33^^,^^36^^,^^40^; 2 on subjects that included adolescents and young adults ages 15–24^37^^,^^39^; and 3 on women of reproductive age.^22^^,^^25^^,^^57^ Finally, some evaluations, and studies related to the evaluations, examined the impact of interventions included in a broader program. For example, 1 evaluation^33^ assessed 8 individual interventions that could have contributed to the positive outcome of a school-based intervention.

We identified 14 high-quality evaluations in which the intervention achieved a statistically significant impact on rapid repeat pregnancy or birth up to 24 months after the index birth, or on contraceptive continuation for 24 months.

An additional 3 high-quality evaluations reported that the interventions resulted in positive trends in reducing repeat pregnancies but that the outcomes were not statistically significant (Supplement Table 2). Two were randomized controlled trials and the third used a quasi-experimental design. In contrast, 7 high-quality evaluations reported no impact of the intervention on repeat pregnancies or births (Supplement Table 2). Five of these were randomized controlled trials and 2 used quasi-experimental designs. These evaluations recorded only a minimal or no difference between the intervention and comparison groups' rates of repeat pregnancies or births.

### Most Effective Interventions for Preventing Rapid Repeat Pregnancy Among Adolescents

In our review of high-quality evaluations, interventions that achieved a statistically significant impact on adolescent, rapid repeat pregnancy or birth rates, or on contraceptive continuation rates, fell into the following 5 broad categories (Table 3).

**TABLE 3.** Interventions Achieving Statistically Significant Impact on Rapid Repeat Pregnancy or Birth Among High-Quality Evaluations (n=14)

Intervention Description	Evaluation	Country	Outcome Measured During Postpartum Period	Repeat Pregnancy or Birth Rate		*P* Value
				Intervention	Control	
**Contraceptive Services and Information**						
Proactive monitoring of contraceptive use, contraceptive education, and inclusion of partner and families	Sullivan 1992^18^	US	Pregnancy <18 months	12%	28%	<.003
Proactive monitoring of contraceptive use, contraceptive education, and inclusion of partner and families	Rabin 1991^36^	US	Pregnancy over 9 years	9%	70%	<.001
**Postpartum Contraceptive Services**						
Postpartum check-ups and provision of contraceptive services within 2 months of index birth in school setting	Seitz 1993^33^	US	Birth <24 months	12%	36%	<.005
Education on the use of LAM and, for intervention group participants only, education on the use of EC in the event of unprotected intercourse and provision of take-home supply of EC	Shaaban 2013^22^	Egypt	Pregnancy <6 months	0.3%^a^	5%	<.001
Education on the use of LAM and support/increased messaging to transition to another modern method by 6 months postpartum (a sub-intervention of a larger birth spacing intervention evaluated by Ahmed 2015^39^)	Ahmed 2015^39^	Bangladesh	Birth <24 months	14%^b^	17%^b^	<.01
**Planning Interventions**						
Preparation of contraceptive plan in the antenatal period (a sub-intervention of a larger pregnancy spacing intervention evaluated by Olds 2002^24^)	Gray 2006 study^17^ (secondary analysis of Olds 2002^24^)	US	Pregnancy 13–24 months	–^c^	–^c^	–^c^
Home visitation by nurses to help women plan the timing of the next pregnancy, rather than avoid unintended pregnancies	Olds 2002^24^	US	Pregnancy <24 months	29%	41%	<.02
Home visitation by nurses to help women plan the timing of the next pregnancy, rather than avoid unintended pregnancies	Kitzman 1997^25^	US	Pregnancy <24 months	36%	47%	<.01
Training adolescents in “if-then” planning for oral contraceptive use	Martin 2011^21^	UK	Pregnancy <24 months	7%	12%	<.02
**Community-Based Social and Behavioral Change Communication**						
Education on postpartum fertility return before return of menses. This was a sub-intervention of birth spacing intervention evaluated by Ahmed 2015.	Cooper 2014 study^16^ (analysis of sub-intervention carried out in Ahmed 2015^39^)	Bangladesh	Birth <24 months	14%^d^	17%	<.01
Interpersonal counseling and community education on the benefits of healthy pregnancy spacing and potential consequences of short pregnancy intervals, with a focus on adolescents and young adults ages 15–24	Sebastian 2012^37^	India	Pregnancy at 9 months	10.5%^e^	16.4%	<.01
Group discussions in homes of influentials to promote positive views of contraceptives and encourage discussions with husbands and friends	Kincaid 2000^57^	Bangladesh	Contraceptive continuation over 2.5 years	–^f^	–^f^	–^f^
**Motivating, Mentoring, and Goal Setting**						
Assistance to adolescents to prepare plans for achieving short- and long-term life goals (a sub-intervention of a larger pregnancy spacing intervention evaluated by Olds 2002^24^)	Gray 2006 study^17^ (secondary analysis of Olds 2002^24^)	US	Pregnancy 7–12 months	–^c^	–^c^	–^c^
Use of mentorship curriculum by women from the community who made home visits to postpartum adolescents every 2 weeks until infant's first birthday	Black 2006^19^	US	Birth <24 months	11%	24%	<.05
Cell phone counseling emphasizing teens' own goals and needs, positive youth assets, healthy relationships, and positive reproductive health practices	Katz 2011^23^	US	Pregnancy <24 months	26%^g^	39%^g^	<.01
Motivational interviewing of adolescents, emphasizing personal goals and self-efficacy	Barnet 2009^20^	US	Birth <24 months	–^h^	–^h^	–^h^
Provision of skills training and job placement for adolescent mothers over age 16 and educational support for mothers under age 16	Drayton 2000^40^	Jamaica	Pregnancy over 4 years	37%	60%	<.05

Abbreviations: EC, emergency contraception; LAM, Lactational Amenorrhea Method.

a Shaaban 2013 reported 2 pregnancies among 579 participants in the intervention group, for a pregnancy rate of 0.3%. The article reported a pregnancy rate of 0.8%, but it is likely a transcription error.

b At 3 months postpartum, contraceptive use was 36% (of which 23% was LAM use) in the intervention group compared with 11% (with no LAM use) in the comparison group. In the intervention group, in part due to LAM users' transition to another method at 6 months postpartum, contraceptive use remained significantly higher in the intervention group than the comparison group at 24 months postpartum (46% vs. 35%, respectively; *P*<.001).

c The study indicated that adolescents with a prenatal contraceptive plan were significantly less likely to conceive at 13–24 months postpartum than adolescents without a plan. 18.6% of adolescents who prepared such a plan did not conceive by 13-24 months, while 0% of those who conceived by 13-24 months had prepared a prenatal contraceptive plan (*P*<.005). Adolescents who formulated short- and long-term goals were significantly less likely to conceive at 7–12 months postpartum than those who did not formulate such goals (*P*<.05).

d Sub-intervention analyzed in Cooper 2014^16^ focused on improving knowledge of postpartum fertility return. The analysis found that 98% of women knew fertility could return before return of menses, and women stated this information motivated them to begin using contraceptives.

e 93% of those in the intervention group reported counseling on use of spacing methods after delivery, whereas 69% of those in the control group reported such counseling (*P*<.01). Women in the intervention group who knew at least 2 spacing messages and at least 2 spacing methods were more likely to adopt a modern method postpartum (*P*<.05).

f Outcome measured was contraceptive continuation for 2.5 years at any point in a woman's life, not necessarily during the postpartum period. In the intervention group, contraceptive continuation for 2.5 years was 43.9% vs. 25.5% in the comparison group (*P*<.001).

g Among adolescents ages 15–17 years.

h Controlling for baseline difference, adolescents who received motivational interviews and home visits were more likely to defer a repeat birth than those in the control group (hazards ratio, 0.4; *P*<.05).

**Contraceptive services coupled with education about modern contraceptive methods and reproductive health:** Comprehensive health and social services with strong emphasis on contraceptive services^18^^,^^36^ and inclusion of partners and families in the contraceptive education activities.^16^^,^^18^^,^^36^^,^^37^^,^^39^^,^^57^ Such services were provided for either postpartum adolescents or for non-postpartum adolescents and young adult parents.

**Postpartum contraceptive services:** Postpartum check-ups and contraceptive service provision within 2 months postpartum in a school setting^33^; education about the use of LAM and the need to transition to another modern method of contraception at 6 months postpartum^39^; and education about LAM and provision of 1 package of emergency contraceptive pills and training on their use should unprotected intercourse occur while practicing LAM when 1 of the 3 LAM conditions was not met.^22^ (WHO classifies LAM as a modern contraceptive method.^58^) The 3 conditions that must be met for LAM use to effectively to protect against pregnancy are: (1) the woman is fully or almost fully breastfeeding, (2) menses have not returned, and (3) the baby is less than 6 months old.

**Planning interventions:** Program emphasis on “planning the next pregnancy,” rather than on avoiding unintended pregnancy^24^^,^^25^; preparation by adolescents of a contraceptive plan (in the antenatal or postnatal period)^17^^,^^24^; and training adolescents in “if-then” planning to facilitate effective use of oral contraceptives.^21^

**Community-based social and behavioral change communication:** Interpersonal counseling and community education on the possibility of postpartum fertility return before the return of menses, and the importance of using contraceptives before menses return to prevent unintended pregnancy^16^^,^^39^; interpersonal counseling and community education on the benefits of healthy pregnancy spacing and the use of contraceptives to prevent adverse outcomes associated with closely spaced births^16^^,^^37^^,^^39^; and group discussions in homes of village influentials to encourage positive views of contraceptives and the use of communication skills to share learning with husbands.^57^

**Motivating, mentoring, and goal-setting interventions:** Preparation of plans by adolescents to achieve short–term life goals (e.g., improved parenting) and long-term goals (e.g., education)^17^^,^^24^; use of a mentorship curriculum by women from the community who presented themselves as “big sisters” to adolescent mothers during home visits^19^; motivational interviewing of adolescents, a counseling style that emphasizes an individual's goals and self-efficacy in relation to complex health behaviors and aims to promote the individual's intention to change^20^; use of a cell phone counseling approach that incorporated aspects of youth asset development models and emphasized teens' own goals and needs, communication skills, and connections with school and adult role models^23^; and skills training and job placement for adolescents over age 16 and educational support for mothers under age 16.^40^

### Less Effective Interventions for Preventing Rapid Repeat Pregnancy Among Adolescents

Seven high-quality evaluations found that the intervention did not achieve a statistically significant impact on rapid repeat pregnancy or births. Four of these were home-based interventions such as home visitation or family support services.^28^^–^^30^^,^^32^ One was a cash transfer to female heads of households,^43^ one was a peer education and support/monetary incentive intervention,^31^ and one was a prenatal education program.^44^ All but two^29^^,^^43^ focused exclusively on adolescents. Five evaluations were randomized controlled trials^28^^–^^32^ and two were quasi-experimental designs.^43^^,^^44^ The less effective interventions helped to highlight design and implementation flaws, which we have included in the lessons.

### Magnitude of Effect on Repeat Pregnancy Rates

We examined the magnitude of effect of selected interventions on repeat pregnancy rates, as reported by 6 high-quality evaluations. The evaluations measured repeat pregnancy or birth at similar time periods (Table 4). While all 6 evaluations reported statistically significant effects, some intervention impacts were greater than others, highlighting the importance of going beyond statistical significance in considering impact on target populations and underscoring the importance of assessing implementation factors that may reduce intervention effectiveness.

**TABLE 4.** Magnitude of Effect on Repeat Pregnancy or Birth Among High-Quality Evaluations Measuring Similar Outcomes at Similar Time Periods^a^ (n=6)

Evaluation	Intervention Description	Outcome Measured During Postpartum Period	Repeat Pregnancy Rates		*P* Value
			Intervention	Control	
**Higher Magnitude of Effect**					
Sullivan 1992^18^	Health care model delivered at teen baby clinic for teen mothers, including social workers, pediatrician, and referral for contraceptive service provision; focused on prevention of repeat pregnancy, return to school, immunizations, and reduced use of emergency room.	Pregnancy <18 months	12%	28%	<.003
Black 2006^19^	Postpartum home-visitation mentoring intervention; curriculum delivered every other week until infant's first birthday by women from community who served as mentors.	Birth <24 months	11%	24%	<.05
Martin 2011^21^	Training for adolescents in “implementation intention formation” (if-then planning) in relation to use of contraceptives.	Pregnancy <24 months	7%	12%	<.02
**Lower Magnitude of Effect**					
Katz 2011^23^	Intensive cell phone counseling intervention to prevent subsequent teen pregnancies by strengthening healthy relationships, reproductive practices, positive youth assets, and teen's own goals and needs.	Pregnancy <24 months	26%^b^	39%^b^	<.01
Olds 2002^24^	Nurse home-visitation intervention to improve health behaviors, prevent rapid repeat pregnancies, improve parent care of children, and maternal life-course development.	Pregnancy <24 months	29%	41%	<.02
Kitzman 1997^25^	Home visitation by nurses to improve newborn and child health and mental development, and to prevent injuries and rapid repeat pregnancies.	Pregnancy <24 months	36%	47%	<.006

a All 6 evaluations were randomized controlled trials and reported statistically significant impact of the intervention on rapid repeat pregnancy or birth rates. All were conducted in the United States, except Martin (2011),^21^ which was conducted in the United Kingdom.

b Among mothers ages 15–17 years.

For example, in 3 high-quality evaluations, at 18–24 months postpartum, the repeat pregnancy or birth rates in the intervention groups were relatively low, ranging from 7% to 12%, compared with repeat pregnancy or birth rates in the comparison groups at 12% to 28%. However, in 3 other high-quality evaluations, the interventions achieved a statistically significant effect on the repeat pregnancy rate, yet the repeat pregnancy rate in the intervention groups was still relatively high, at 26% to 36% and 39% to 47% in the comparison groups.

All 3 of the evaluations that showed a lower magnitude of effect reported challenges that may have influenced outcomes. For example, the evaluation of a cell phone intervention^23^ reported that the adolescents “would not always answer calls” for scheduled counseling sessions, and some teens lost or damaged their phones. The Olds 2002 evaluation^24^ of a home visitation intervention reported that 40% of the subjects, after review and testing, were characterized as having “low psychological resources.” The Kitzman 1997 evaluation^25^ of a home visitation program reported that all subjects had at least 2 sociodemographic risk characteristics such as being unmarried, having less than 12 years of education, or being unemployed.

We also note that the magnitude of the effect may be greater in populations where the repeat pregnancy rate is already lower; compare the repeat pregnancy rate in the comparison groups of the evaluations showing a higher magnitude of effect (20% to 30%) with the repeat pregnancy rate of the intervention groups of the evaluations showing a lower magnitude of effect (26% to 36%). This could suggest that the success of a specific intervention may be partially dependent on the broader program environment, including norms around adolescent childbearing and contraceptive use.

### Program Design and Implementation Lessons

Based only on the 24 high-quality evaluations included in our review, we identified 5 program design and implementation lessons about interventions that are linked with prevention of rapid repeat pregnancy.

We identified 5 program design and implementation lessons from interventions that are linked with statistically significant reductions in rapid repeat pregnancy.

#### Proactive Program Monitoring of Contraceptive Use, Providing Contraceptive Education, and Involving Partners and Families Are Linked to Reductions in Rapid Repeat Pregnancy

Three evaluations^18^^,^^28^^,^^36^ emphasized the importance of proactive program monitoring of adolescents' contraceptive use and contraceptive education. These evaluations observed that, in comprehensive programs that work across sectors and disciplines, the contraceptive service delivery component must be well-designed, easily accessible, well-implemented, and closely monitored. Programs should include quality counseling, method provision, and services by trained providers at the time that services are requested. These may seem like rather obvious activities to be included in interventions to reduce repeat pregnancies, but we found a distinct lack of attention to contraceptive services in a number of evaluations of comprehensive service programs. In addition, 5 evaluations^18^^,^^36^^,^^37^^,^^39^^,^^57^ and 1 study^16^ stressed inclusion of partners and families in program activities.

In interventions that achieved impact, providers paid intense attention to educating the adolescents and their partners and families about contraceptives, and proactively monitored contraceptive use. For example, one evaluation of a comprehensive health care program for adolescent mothers^18^ was carried out in a well-baby clinic for teen mothers and staffed by a nurse practitioner, a pediatrician, and a social worker. Key goals were prevention of repeat pregnancy and the mother's return to school. The evaluation observed that the providers were proactive—”all three providers tracked contraceptive use, satisfaction with the method, referral for a different method, and engaged in active follow-up if appointments were missed.” Providers wrote notes in subjects' charts on “whether the mother was using family planning and whether she liked her method.” The providers insisted on “talking with the mother about her plans for the future,” along with her use of family planning, and focused on the mother's plans to return to school. The program managers urged that entire families be involved in these discussions because “chang(ing) attitudes about the future will do more to delay these pregnancies than working only with the adolescent mother.”^18^ At 18 months postpartum, the repeat pregnancy rate among the intervention group was 12% (13/108) compared with 28% (32/113) among the comparison group (*P*<.003).

Another evaluation assessed a comprehensive, experimental prenatal and family planning program carried out by a multidisciplinary hospital team consisting of a gynecologist, pediatrician, social worker, and health educator.^36^ The experimental program included a reproductive health and family life education program for the mother, her partner, and family every other week, and emphasized program attendance. Over 75% of the intervention group participated regularly; in contrast, only 18% of the control group attended similar activities offered by routine services. The evaluators reported that “primary pregnancy prevention efforts (sexual education) and secondary efforts (the importance of sexual responsibility and contraceptive education, availability, and utilization) are important front-line strategies …” Critical activities to prevent second pregnancies included “mobilization of the partner, family, teachers, and social support systems to … engage in a dialogue which stresses sexual education and contraceptive responsibility.” Over the 9-year implementation period, 9% of intervention group participants experienced a repeat pregnancy, while 70% of comparison group participants did (*P*<.001). Contraceptive use in the intervention group was 85% compared with 22% in the control group (*P*<.001).

In a community-based intervention that did not achieve impact, trained home visitors provided services to adolescent parents until the index child was 2 years old.^28^ They delivered a parenting curriculum, encouraged contraceptive use, connected the teen with primary care, and promoted school continuation. The program linked teens with primary care physicians, but program managers “did not assess the content of primary care … consequently we were unable to determine whether primary care physicians provided appropriate contraceptive services.” At 24 months after the index birth, the repeat pregnancy rate was 45% in the intervention group and 38% in the comparison group.

#### Providing Postpartum Mothers Contraceptive Counseling and Services Soon After Delivery Are Linked to Reductions in Rapid Repeat Pregnancy

Four evaluations provide evidence to support this lesson.^22^^,^^29^^,^^33^^,^^39^ An evaluation of a public school-based intervention found that teen mothers who spent longer than 7 weeks attending a special school for adolescent parents before returning to their regular school were much less likely to have had a second child over the next 5 years, compared with teen mothers who returned to their regular school less than 7 weeks after delivery.^33^ The authors analyzed 8 potential causal mechanisms that might have contributed to this result, but only 2 were statistically significant: avoidance of sexual activity and a “postpartum check-up before exiting” the special school. Those who attended the school longer than 7 weeks were required to have a postpartum check-up, which took place within 2 months of delivery. During the check-up, the new mothers received a contraceptive counseling session, at which almost three-quarters of participants accepted injectable contraceptives. Within 2 years of the index birth, only 12% (6/50) of students who received the postpartum check-up delivered a second child, compared with 36% (19/52) of students who did not receive a check-up (*P*<.005).

In a community-based, home visitation intervention in Bangladesh,^39^ 10th-grade-level community health workers (CHWs) educated postpartum women on the use of LAM and the importance of transitioning to another modern method at 6 months postpartum. In addition, “LAM Ambassadors” (practicing LAM users with their healthy infants) served as role models and actively promoted LAM as an immediate post-delivery contraceptive method particularly appropriate for this rural area. The evaluation found that “a major increase in contraceptive use in the early postpartum period was attributable to a higher use of LAM in the intervention area.” At 3 months postpartum, contraceptive use was 36% in the intervention group (of which 23% reported use of LAM) and 11% in the comparison group (*P*<.001). Women in the comparison area did not report use of LAM during any survey round. In part due to subjects' transition from LAM to another modern method at 6 months, contraceptive prevalence remained significantly higher in the intervention area at 24 months after the index birth (46% versus 35%, respectively; *P*<.001). In addition, the rate of reporting a short birth interval of less than 24 months was significantly lower (*P*<.01) in the intervention area (14%) than in the comparison area (17%).

An evaluation of a clinic-based intervention in Egypt described how pregnant intervention group clients were counseled on use of LAM and use of emergency contraception if unprotected intercourse occurred when 1 of the 3 LAM conditions was not met.^22^ Intervention group clients were given 1 packet of emergency contraceptive pills to take home while the comparison group received only counseling about LAM. Among the intervention group, 44% used emergency contraception, while none in the comparison group used it. At 6 months postpartum, the repeat pregnancy rate was only 0.3% (2/579) in the intervention group, compared with 5% (29/579) in the comparison group (*P*<.001). In addition, significantly more women in the intervention group initiated regular contraception within or shortly after 6 months postpartum than those in the comparison group (30.5% vs. 7.3%, respectively; *P*<.001).

In contrast, an evaluation of a U.S. home visitation program that did not achieve impact found that, at 24 months after enrollment, 21% (29/141) of intervention group participants and 20% (22/112) of control group participants experienced repeat pregnancies.^29^ The evaluation noted that the “lack of program effects can be traced to the program's design and implementation.” The program required only that family planning be introduced any time during a family's first year of enrollment. The authors observed “(B)ecause conception can occur very soon after an index birth … a better design would be to introduce family planning counseling early in a family's enrollment in home visiting.”

#### Helping Adolescents Plan for the Next Pregnancy, Plan Contraceptive Use, or Prepare a Contraceptive Plan Is Linked to Reductions in Rapid Repeat Pregnancy

Three evaluations^21^^,^^24^^,^^25^ and 1 study^17^ provide evidence to support this lesson. The evaluations identified 3 contraceptive planning interventions that were designed to help women and girls plan to avoid rapid repeat pregnancies:

- Training in planning to use contraceptives^21^
- Planning the timing of the next pregnancy, rather than trying to prevent unintended pregnancy^24^^,^^25^
- Preparing a contraceptive plan^17^

A contraceptive plan is intended to help women and girls clarify and act on their reproductive intentions and make an informed choice about contraceptive use to achieve their reproductive life goals. (In 2006, the U.S. Centers for Disease Control and Prevention, in its guidelines for improved preconception care, issued a recommendation encouraging all women, men, and couples to prepare a reproductive life plan, to avoid unintended pregnancies and reduce adverse pregnancy outcomes.^59^)

**Figure:**
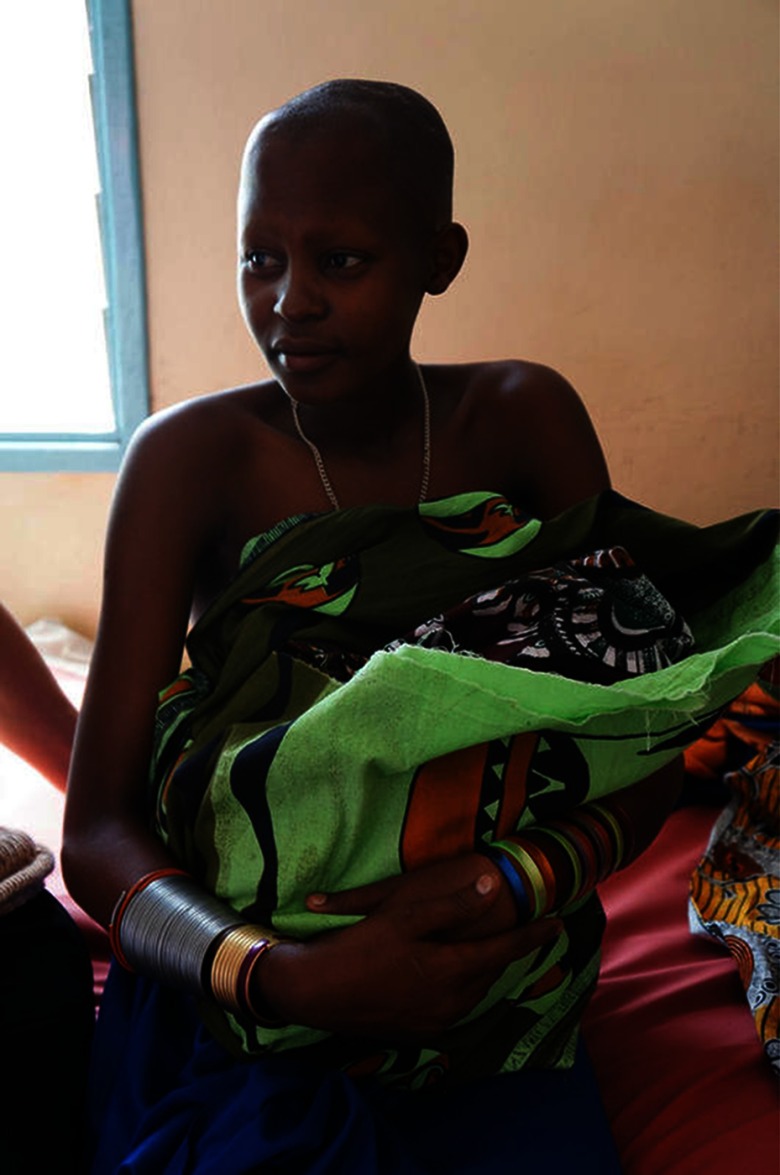
A 16-year-old girl holds her first child at a district health facility in Tanzania. © 2014 Megan Ivankovich/WI-HER LLC, Courtesy of Photoshare

One evaluation assessed the effect of an intervention that assisted adolescent girls in practicing “implementation intention formation” or “if-then planning.”^21^ This is a planning approach that specifies in advance the “when, where, and how” of behaviors involved in contraceptive use. (For example, “If I am in the bathroom after brushing my teeth in the morning, then I will take my contraceptive pill!”) The evaluation found that this intervention was effective in helping adolescent girls use contraception for 2 years, and significantly reduced the percentage of intervention group participants who received a positive pregnancy test (7%, or 8/112) compared with comparison group participants (12%, or 14/115) at the 2-year follow up (*P*<.02).

Two evaluations assessed similarly designed nurse home visitation programs targeted to low-income mothers.^24^^,^^25^ The theoretical foundations of the home visitation model reflect the importance of enhancing mothers' self-efficacy.^60^ Specifically, the curricula-based, home visitation intervention aimed to improve pregnancy outcomes, parental caregiving, and maternal life-course development (defined as helping women return to work or school and to plan future pregnancies). The emphasis was on the women's desired timing of the next pregnancy, rather than on avoiding unintended pregnancies. In both interventions, nurses made an average of 7 home visits during pregnancy and 26 visits up to the index child's second birthday. The nurses “encouraged women to clarify plans for completing their education, returning to work, and bearing additional children,” aiming to help women achieve what they considered their optimal family size.^61^ At 24 months postpartum in both programs, women who had received visits by nurses were significantly less likely to have had a subsequent pregnancy than women in the control group (29% vs. 41%, respectively; *P*<.02 in the Olds 2002 evaluation^24^; 36% vs. 47%, respectively; *P*<.01 in the Kitzman 1997 evaluation^25^).

The home visitation intervention evaluated by Olds 2002 achieved a statistically significant effect, yet the magnitude of effect was relatively low (repeat pregnancy rate, 29% intervention vs. 41% control; *P*<.02).^24^ A study by Gray (2006)^17^ of the data generated by the Olds 2002 evaluation^24^ asked what could have been done to achieve greater impact. Gray found that having an antenatal contraceptive plan was significantly associated with not conceiving at 13–24 months postpartum (*P*<.005).^17^ But few program participants reported having a contraceptive plan. Of the 29% of adolescents who reported a subsequent pregnancy by 24 months postpartum, *none* had prepared an antenatal contraceptive plan. In contrast, 19% of those who were not pregnant had prepared such a plan (*P*<.01).^17^ Gray notes that “the nurses rarely documented that they explicitly tried to help the teens postpone a second pregnancy. Assistance … that might motivate the teen to keep using birth control were only recorded during 30 percent of the visits.”^17^

#### Enhancing Understanding of Contraceptives' Role in Determining Positive Life Outcomes Is Linked to Reductions in Rapid Repeat Pregnancy

Three evaluations^37^^,^^39^^,^^57^ and 1 study^16^ provide evidence to support this lesson. The interventions described in these evaluations were designed to change the way that clients thought about contraception—that is, rather than focusing on side effects, for example, intervention messages were designed to help clients understand the role that contraceptives could play in determining positive life outcomes. The evaluations showed that such messages were strongly linked with increased use of contraceptives.

For example, one evaluation examined a community-based, social networks approach that diffused new and positive ideas related to contraception across communities and social networks, and assessed contraceptive use and continuation over a period of 2.5 years.^57^ In the intervention, government field workers were trained to organize village-level group discussions with women in the homes of opinion leaders and facilitate the development of positive attitudes toward contraceptives. Group discussions focused on 4 messages: (1) practicing family planning to have fewer children may help your family avoid poverty; (2) couples that practice family planning are better able to provide food for their children; (3) having fewer children helps families to raise them properly; and (4) practicing family planning improves the relationship between a husband and a wife. It was anticipated that holding discussions in the homes of village opinion leaders would provide greater opportunities for validation and support of these ideas, compared with field workers' home visits with individual women. After 2 years, overall ideation (e.g., knowledge of and support for new ideas and practices related to contraceptives) increased by 0.79 points among social networks participants and by 0.39 points among women with home visits, and declined 1.32 points among women having no contact with government field workers. Over 2 years, contraceptive prevalence increased 4.7% among social networks participants and 0.9% among women who continued to be visited at home, and declined 9.4% among women with no health worker contact at all. The evaluation concluded that the strong impact of the social networks intervention on contraceptive use was due primarily to its impact on contraceptive continuation: the family planning continuation rate at 2.5 years was 43.9% for social networks participants, 25.5% for women who received home visits, and 6.7% for women with no health worker contact (*P*<.001).^57^

An evaluation in Uttar Pradesh, India, assessed a community-based behavior change communication intervention.^37^ CHWs were trained to educate mothers (with a focus on women ages 15–24 years) and mothers-in-law using leaflets, posters, and wall hangings on postpartum care and healthy timing and spacing of pregnancy, advising them to wait at least 24 months after a live birth before conceiving again. At the pre-test, 14% of CHWs answered all test questions correctly except the one on the 3 conditions of LAM, while 95% answered all test questions correctly (including the LAM question) in the post-test. After 5 months of implementation, adolescents and young adults achieved significantly increased knowledge about 6 of 8 variables (*P*<.05):

- IUD is placed in the uterus and is effective for 10 years
- Correct use of emergency contraception
- Three conditions that must be met for effective use of LAM
- Three adverse outcomes of short-interval pregnancies
- Health of woman, last child, and fetus are affected by closely spaced pregnancies
- Correct condom use

Those with greater knowledge of at least 2 healthy spacing messages (*P*<.05) and correct knowledge of methods (*P*<.01) were more likely to adopt a modern contraceptive method. At 9 months postpartum, modern contraceptive use for spacing was 57% in the intervention group and 30% in the comparison group, while the repeat pregnancy rate was 10.5% in the intervention group and 16.4% in the comparison group (*P*<.01).^37^

A study conducted by Cooper (2014) in Bangladesh^16^ reported on the introduction of ideas related to birth spacing and fertility return before menses, in a community-based postpartum program.^39^ This social and behavior change communication intervention was designed to help women, men, and mothers-in-law understand that fertility could return before menses return; use of contraception before menses return may prevent an unintended pregnancy; and newborn health is improved with spacing pregnancies at least 24 months after the preceding live birth. (After delivery, among non-breastfeeding women, ovulation may occur at approximately 6 weeks and, for some women, as early as 3 weeks.^62^) The Cooper study, based on 40 in-depth interviews, found almost universal exposure to information about the return of fertility before menses, with 97.5% of the women recognizing that a woman could become pregnant prior to menses return. Interviews revealed “thirty-five of forty respondents reported that this information led them to make a change in their behavior” and begin using postpartum contraception. In addition, 58% of women understood that 3-year birth intervals were healthy. No respondent, including men and mothers-in-law, expressed the view that women should conceive less than 2 years after the index birth. At 24 months postpartum, the modern contraceptive prevalence rate was significantly higher among the intervention group than the control group (46% vs. 35%; *P*<.001), and short birth intervals of less than 24 months significantly lower (14% vs. 17%, respectively; *P*<.01).

An evaluation examining the effect of monetary incentives and peer group discussions on repeat adolescent pregnancies in 3 treatment groups and 1 control group illustrates the importance of ensuring positive messages.^31^ The evaluation found that monetary incentives draw the teens to the sites where they could discuss use of contraception, but the peer group discussions did not prevent repeat pregnancies. The evaluation commented that “at times one participant would hear another talking about the benefits of having another child … thereby reinforcing and validating the very practices and thinking patterns the groups were designed to extinguish.” In this intervention, on average, 39% in all groups experienced pregnancy within 24 months of delivery of the index child.

#### Mentoring, Motivating, and Goal Setting Are Linked to Reductions in Rapid Repeat Pregnancy

Three evaluations^19^^,^^20^^,^^23^ and 1 study^17^ showed that curriculum-based interventions that include motivational, mentoring, and goal-setting elements can positively influence rates of repeat pregnancy.

One evaluation examined a home visit mentorship and curriculum-based intervention for African-American teen mothers delivered every other week until the index infant's first birthday by college-educated, African-American, single mothers who presented themselves as “big sisters.”^19^ This mentoring intervention stressed negotiation skills, personal development, and parenting. The mentors emphasized “personal values and decision-making regarding subsequent pregnancies, access to birth control, and goal setting” rather than overt messaging on avoiding a second birth. Having at least 2 home visits increased the likelihood of not having a second child by more than threefold (odds ratio 3.3; 95% confidence interval, 3.0 to 5.1). At 24 months postpartum, 11% (8/70) of intervention group participants experienced repeat births compared with 24% (19/79) of the control group (*P*<.05). There were no second births at 24 months postpartum among women who attended 8 sessions.

Another evaluation assessed an intervention called “motivational interviewing” combined with home visiting.^20^ Motivational interviewing is a counseling style that emphasizes an individual's goals, using a tool called CAMI (Computer-Assisted Motivational Interviewing). The evaluation notes that “motivational interviewing aims to highlight the discrepancies between current behaviors and personal goals and self-efficacy, in relation to complex behaviors.” At 24 months postpartum, mothers who received at least 2 CAMI sessions coupled with home visitation were significantly less likely to experience a repeat birth (13.8%, or 11/80) than participants in the usual care group (25%, or 17/68) (*P*<.05).

A cell phone counseling intervention randomized adolescent subjects to a cell phone counseling group and usual care.^23^ The intervention aimed to strengthen healthy relationships, improve reproductive practices, and prevent second pregnancies while emphasizing positive youth assets. Trained counselors scheduled 35–45-minute phone sessions once a week for the first 6 months postpartum and then every 2 weeks over the next 12 months. Curriculum content emphasized building knowledge of health risks and developing positive teen attitudes and skills while emphasizing the teens' own goals and needs. The curriculum also addressed improving partner communication and negotiation skills and resisting peer pressure for risk behaviors. Among mothers 15–17 years, the rate of subsequent pregnancy was 26% in the intervention group and 39% in the usual care group (*P*<.01). In the 15–17-year-old age group, increasing treatment intensity was associated with longer time to subsequent pregnancy.

A study by Gray (2006)^17^ of data generated in a high-quality home visitation evaluation^24^ found that adolescent participants' formulation of short- and long-term educational goals was significantly associated with not conceiving at 0–6 months postpartum (*P*<.001) and at 7–12 months postpartum (*P*<.05).

An evaluation of an intensive home visitation intervention that did *not* achieve impact observed that a key shortcoming was lack of goal setting.^29^ The evaluation found that there was “no evidence that family planning was linked to motivating the parents to avoid rapid, repeat births to achieve personal life goals and to promote effective parenting of the index child.” There was “no evidence of protocols for addressing fertility and for relating subsequent births to parents' abilities to achieve their personal goals for life course development.” At 1-year follow up, the repeat birth rates in the intervention and control groups were 21% and 20%, respectively.

Gray (2006)^17^ found that goal setting was strongly linked with not conceiving. In the Olds 2002 intervention,^24^ however, while almost all teens (94.6%) developed short-term goals such as returning to school, only 20% made efforts “toward a long-term goal, such as developing a 4–5 year contraceptive and work-study plan.” In this intervention, the repeat pregnancy rate in the intervention group, although statistically significant, was still quite high at 29% compared with 41% in the comparison group (*P*<.02).

#### Additional Factors That Influence Rapid Repeat Pregnancy

Additional factors identified in the high-quality evaluations included in this review that reduced the effectiveness of the interventions included:

- Depression^23^^,^^28^
- Reduced program intensity (e.g., a postpartum program ended 1 year early)^26^
- Cultural factors (e.g., in Bangladesh, husbands of intervention group subjects worked in the Middle East and it was unacceptable for a woman to use contraceptives while the husband was away)^39^
- Lack of male and family involvement^30^
- Lack of shared goals (i.e., program participants did not share the goal of preventing a second birth)^32^

## DISCUSSION

This review demonstrates that well-designed and implemented interventions can reduce rapid repeat pregnancy among adolescents. We identified 14 high-quality evaluations of interventions that achieved a statistically significant reduction of postpartum repeat pregnancy or birth rates, or increased contraceptive continuation for at least 2 years. The interventions assessed in these high-quality evaluations fell into the following 5 broad categories:

- Provision of contraceptive services, monitoring contraceptive use, provision of contraceptive education, and inclusion of partners and families
- Postpartum counseling and contraceptive services provided soon after delivery
- Pregnancy or contraceptive use planning interventions
- Community-based social and behavioral change communication interventions that help adolescents understand the role that contraceptives can play in determining positive life outcomes
- Motivating, mentoring, and goal-setting interventions

We are not recommending that all 5 types of interventions that were shown to be effective be implemented simultaneously. However, recognizing potentially synergystic effects, we do recommend testing various combinations of these interventions, with access to contraception as the foundational activity. Experience from the field (and not yet necessarily reflected in the published literature) recognizes the value of a socioecological approach to adolescent pregnancy prevention that intervenes at the individual, family, and community level. We anticipate that some combination of these interventions may create both individual motivation and family/community support for pregnancy spacing. These interventions may also be effective in preventing the first adolescent pregnancy or induced abortion, and as part of postabortion care activities to prevent repeat abortion. The recommended interventions should be tested as part of activities to achieve these outcomes.

A recent global review conducted by the YouthPower project found 5 life/soft skills contribute significantly to adolescents' ability to engage in healthy behaviors that lead to positive sexual and reproductive health outcomes—goal orientation, positive self-concept, self-control, higher-order thinking, and communications skills.^63^ Indeed, the high-quality evaluations of interventions in this review addressed the development of most of these skills across the range of study populations, such as helping adolescents develop contraceptive plans and short- and long-term plans (goal setting); strengthening engagement and communication with husbands, partners, and families (communication); and understanding the health implications for their newborn of closely spaced births (higher-order thinking skills).

A key finding of our review is that effective interventions to prevent rapid repeat pregnancy link adolescent-friendly clinical contraceptive services *with non-clinical interventions that contribute to positive youth development*. This could be, for example, an intervention that facilitates access to contraception, helps adolescents plan and envision a future for themselves, and supports the acquisition of life skills and better understanding of the value of contraception for achieving one's life goals. Civil society organizations, with deep knowledge of their communities, could possibly be well-suited to test and adapt the non-clinical, evidence-based approaches identified in this review. However, these activities cannot stand alone and must be aligned with a contraceptive service delivery component, whether clinical or community-based.

Effective interventions to prevent rapid repeat pregnancy link adolescent-friendly clinical contraceptive services with non-clinical interventions that contribute to positive youth development.

Our findings are consistent with the literature that finds that interventions can be effective in improving adolescent cognitive capacities,^64^ i.e., executive functions that encompass an individual's ability to organize thoughts and activities, prioritize tasks, manage time effectively, and make decisions.^65^ This research sees adolescence as a time of risk and opportunity. Because of recent scientific advances, we now know that brain development—with changes in structure and function—occurs well into the twenties. The limbic system and the amygdala, which are responsible for pleasure and excitement seeking, develop ahead of the forebrain, which is responsible for executive functions, including planning, self-management, and impulse control.^66^ As a result, a young person may know and understand the negative consequences of a particular action, such as having unprotected sex or driving under the influence of alcohol, but may not be able to stop him or herself or resist peer pressure to carry out the action. The research recognizes the “plasticity” of the adolescent brain. This characteristic contributes to abilities to learn and adapt new skills during adolescence, thus marking adolescence as a “period of vulnerabilities, but also great opportunities in terms of … interventions.”^64^^,^^67^

Our review also calls to attention the need for continued thinking about the concepts of “intended” and “unintended” pregnancy. In some contexts, a pregnancy may be “intended” by a young woman only because it is socially and culturally expected. On the other hand, it may not be “intended” by her but occurs because she does not have the power to resist community and family social pressures related to childbearing. The individual interventions (counseling, planning, goal setting) and social interventions (community education and influencing norms) discussed in this review may help young women, and their families, become more informed and help change behavioral intentions and behaviors.

### Recommendations for Action

We recommend the following programmatic actions, which we believe are practical, will substantially strengthen the design and implementation of adolescent programs, and can be implemented at scale.

- **Target contraceptive services and information to first-time mothers/parents.** Services should be targeted during antenatal care, as well as before discharge from the delivery facility, during the immediate or early postpartum period, and during childhood immunization visits at 1–2 months postpartum, with a special focus on very young first-time parents (ages 12–15).
- **Convey information that helps adolescents understand the positive role that contraceptives can play in their lives.** Include the messages identified in the evaluations reviewed here (Box), as they are strongly linked with increased contraceptive use and prevention of rapid repeat pregnancy, and test other evidence-based messages as culturally relevant.
- **Help first-time mothers/parents identify their short- and long-term reproductive intentions and prepare contraceptive use plans to achieve those intentions.** Test the effectiveness of using antenatal or postnatal contraceptive plans to help women and girls achieve 2- to 3-year reproductive intentions. Address cultural norms, and involve influential members in the family and community who may limit adolescents' ability to act on their intentions and carry out their plans.
- **Test, adapt, and scale up the 3 community-based interventions included in this review.**^37^^,^^39^^,^^57^ Include their social and behavioral change messages (Box), especially for first-time parents, and for spouses, mothers-in-law, and other persons who are influential in adolescents' lives, as relevant.^68^
- **Test engaging civil society organizations to motivate and mentor new mothers and first-time parents, help set goals, and support the adoption of new social norms and practices.** Such organizations might be able to assist with introducing the messages used in effective programs, and facilitate setting of short- and long-term life goals especially for first-time parents. They might adapt the motivational and goal-setting curricula used in the interventions reviewed in this article. Partnerships are needed between government and civil society to deliver these multicomponent interventions.

BOX Messages Conveyed in High-Quality Evaluations of Interventions Contributing to Prevention of Rapid Repeat Pregnancy or Contraceptive Continuation **Topics of Messages to Prevent Rapid Repeat Pregnancy**

- Intrauterine device is placed in the uterus and is effective for 10 years
- Correct use of emergency contraception
- Promotion of the Lactational Amenorrhea Method (LAM) for first 6 months postpartum; 3 conditions that must be met for effective use of LAM; transition from LAM to another modern contraceptive method
- Benefits of longer birth intervals; adverse outcomes of short-interval births or pregnancies
  - After a live birth, wait at least 24 months before attempting a pregnancy to reduce the risk of adverse maternal, perinatal, and infant outcomes
  - Benefits of healthy timing and spacing of pregnancies are reduced risk of preterm births and small for gestational age, increased chance that infants will experience the health benefits of breastfeeding for full 2 years
- Health of woman, last child, and fetus are affected by closely spaced pregnancies
- Correct condom use
- Essential newborn care, including exclusive breastfeeding
- Timing of, and signs indicating, return to fertility
- Discussion of contraceptive methods, potential side effects, strategies to minimize side effects
- Referral to health facility for contraceptive methods, if needed

**Group Messages to Promote Contraceptive Continuation**

- Practicing family planning to have fewer children may help your family avoid poverty
- Couples that practice family planning are better able to provide food for their children
- Having fewer children helps families to raise them properly
- Practicing family planning improves the relationship between a husband and a wife

Sources: Cooper 2014,^16^ Shaaban 2013,^22^ Sebastian 2012,^37^ Ahmed 2015,^39^ Kincaid 2000,^57^ and Ahmed 2013.^68^

### Limitations

This review has several important limitations and caveats. First, as we limit our conclusions to the findings from high-quality evaluations of effective interventions that show statistical impact on the outcomes of interest, we limit the number of studies from which to draw lessons. However, by using these stringent criteria we are confident in the results. Second, some of the evaluations have small sample sizes (<250 subjects), but other evaluations were based on relatively large samples, some with more than 1,000 subjects. Third, although there were interventions that demonstrated success, analysis of intervention sub-components to better identify the causal pathway to impact was lacking. Caution is needed before implementing, replicating, or taking to scale these “successful” interventions, especially those that were designed for adolescents based in the United States or United Kingdom, in different sociocultural and/or economic settings. At the same time, while translational research is needed, there is evidence that interventions that have been shown to be effective in one setting have been successfully applied in very different settings.^69^ Given the number of high-quality U.S.-based studies in our review, careful attention nevertheless should be paid to their implementation in developing countries.

While not a limitation, it is essential to ensure that interventions are designed to address the heterogeneity of adolescent populations within and between countries as seen in several of the studies. For example, the interventions targeted diverse populations such as young married couples in North India; urban, better-educated adolescents in Jamaica; poor rural women in Northeast Bangladesh; and largely urban, poor populations in the United States and United Kingdom. Clearly, interventions must be tailored differently to the contexts and prevailing social and cultural norms of country and regional settings. In some settings, early marriage is common and nearly all fertility occurs within the context of marriage. In others, adolescents are mostly sexually active outside of a formal union and lack adequate access to contraceptive information and services. In both settings, postpartum contraception should be a focus of interventions, but the approaches used to increase contraceptive uptake will likely differ. In any event, more interventions need to be designed that address the root causes of rapid repeat pregnancy, such as social norms that promote early marriage and inequitable gender norms, as well as missed opportunities (in antenatal and postpartum care) to provide rights-based contraception.

## CONCLUSIONS

Evidence from high-quality evaluations suggests that providing adolescent-friendly contraceptive services, with involvement of partners and influential family members, is critical to preventing rapid repeat pregnancies among adolescents. Linking such clinical contraceptive services with non-clinical activities that build adolescents' life skills, enhance understanding of the positive role that contraceptives can play in determining life outcomes, and provide mentoring and goal setting is an evidence-based approach to preventing rapid adolescent childbearing. As new programs are designed and implemented, it will be important to keep in mind a lesson from one of the studies—that is, to enhance program effectiveness “frame child spacing as a means to an end” while “helping the teens understand that the goal of family planning (is) not (necessarily) to postpone the birth of the next child for two years, but to optimize the chance of obtaining what they most want for themselves in life.”^17^

## Supplementary Material

Supplement Table 1

## Notes

**Peer Reviewed**

**Funding:** USAID funded the analyses provided in Table 1 and Figure 1.
