# Supplementary material for: Interventions for Preventing Unintended, Rapid Repeat Pregnancy Among Adolescents: A Review of the Evidence and Lessons From High-Quality Evaluations
Source: Glob Health Sci Pract. 2017 Dec 28;5(4):547–70. doi: 10.9745/GHSP-D-17-00131 (PMC5752603; doi:10.9745/GHSP-D-17-00131)
Supplement: Supplement Table 1 [file GHSP-D-17-00131_index.html]

Supplement to Interventions for Preventing Unintended, Rapid Repeat Pregnancy Among Adolescents: A Review of the Evidence and Lessons From High-Quality Evaluations | Global Health: Science and Practice

## Supplemental material

- Text s01, DOCX - Text s01, DOCX
- Text s02, DOCX - Text s02, DOCX
