# Supplementary material for: Interventions for Preventing Unintended, Rapid Repeat Pregnancy Among Adolescents: A Review of the Evidence and Lessons From High-Quality Evaluations
Source: Glob Health Sci Pract. 2017 Dec 28;5(4):547–70. doi: 10.9745/GHSP-D-17-00131 (PMC5752603; doi:10.9745/GHSP-D-17-00131)
Supplement: Supplement Table 1 [file 17-00131-Norton-Supplement-Table1.docx]

**SUPPLEMENT TABLE 1.** Quality Assessment of Evaluations Included in the Review (N=40)

|  | **Quality Assessment Criteria** | | | | | |  |
| --- | --- | --- | --- | --- | --- | --- | --- |
| **Evaluation** | **Use of quantitative/**  **qualitative analyses to attribute change to the intervention** | **Randomization of individual subjects** | **Use of concurrent comparison group** | **Sample size >100** | **Baseline and endline evaluation** | **Measured valid and relevant outcome^a^** | **Total score and quality assessment^b^** |
| **Randomized Controlled Trials** | | | | | | | |
| 1 Sullivan 1992^18^ | Y | Y | Y | Y  243 adolescent mothers | Y | Y  Repeat pregnancy at 18 months after index birth | 6  High |
| 2 Black 2006^19^ | Y | Y | Y | Y  181 adolescent mothers, 149 completed the evaluation | Y | Y  Repeat birth at 24 months after index birth | 6  High |
| 3 Barnet 2009^20^ | Y | Y | Y | Y  235 pregnant adolescents | Y | Y  Repeat birth at 24 months after index birth | 6  High |
| 4 Martin 2011^21^ | Y | Y | Y | Y  227 adolescents | Y | Y  Repeat pregnancy at 24 months after index birth | 6  High |
| 5 Shaaban 2013^22^ | Y | Y | Y | Y  1158 postpartum women | Y | Y  Repeat pregnancy after LAM and EC use at 6 months after index birth | 6  High |
| 6 Katz 2011^23^ | Y | Y | Y | Y  249 adolescents | Y | Y  Repeat pregnancy at  24 months after index birth | 6  High |
| 7 Olds 2002^24^ | Y | Y | Y | Y  735 women who had no previous births | Y | Y  Repeat pregnancy at 24 months after index birth | 6  High |
| 8 Kitzman 1997^25^ | Y | Y | Y | Y  1139 women | Y | Y  Repeat pregnancy at 24 months after index birth | 6  High |
| 9 Koniak-Griffen 2003^26^ | Y | Y | Y | Y  101 adolescent mothers | Y | Y  Repeat pregnancy at 24 months after index birth | 6  High |
| 10 Ford 2002^27^ | Y | Y | Y | Y  282 pregnant adolescents | Y | Y  Repeat pregnancy at 1 year after index birth | 6  High |
| 11 Barnet 2007^28^ | Y | Y | Y | N  84 adolescents | Y | Y  Repeat pregnancy at 12 and 24 months after index birth | 5  High |
| 12 El-Kamary 2004^29^ | Y | Y | Y | Y  643 families | Y | Y  Repeat pregnancy at 1, 2, 3 years after enrollment into the program | 6  High |
| 13 Cherniss 1996^30^ | Y | Y | Y | N  94 adolescent mothers  (originally 116) | Y | Y  Repeat pregnancy at 12 and 24 months after index birth | 5  High |
| 14 Stevens-Simon 1997^31^ | Y | Y | Y | Y  286 primiparous adolescent mothers <age 18 | Y | Y  Repeat pregnancy at 6, 12, 18, and 24 months after index birth | 6  High |
| 15 Sims 2002^32^ | Y | Y  Randomized to 2 different treatment groups | Y | N  99 adolescents | Y | Y  Repeat  pregnancy at 24 months after index birth | 5  High |
| **QUASI-EXPERIMENTAL DESIGNS** | | | | | | | |
| 1 Seitz 1993^33^ | Y | N | Y | Y  102 women less than age 19 on delivery date | Y | Y  Repeat birth by 24 months and 6 years after index birth | 5  High |
| 2 Key 2008^34^ | Y | N  Comparison group subjects randomly selected from state birth certificate data | N | Y  63 intervention adolescents  252 comparison group | Y | Y  Repeat birth by 24 months after index birth or until age 20 | 4  Moderate |
| 3 Solomon 1998^35^ | Y | N | Y | N  63 adolescent mothers | Y | Y  Repeat pregnancy 2 and 3 years after enrollment | 4  Moderate |
| 4 Rabin 1991^36^ | Y | N | Y | Y  498 intervention adolescents  91 control adolescents | Y | Y  Repeat pregnancy over 9-year implementation period | 5  High |
| 5 Sebastian 2012^37^ | Y | N  Cluster randomized | Y | Y  959 pregnant women aged 15–24 | Y | Y  Repeat pregnancy by 9 months after index birth | 5  High |
| 6 Lewis 2012^38^ | Y | N | Y | Y  144  adolescent mothers | Y | Y  Repeat births within 3 years of index birth | 5  High |
| 7 Ahmed 2015^39^ | Y | N | Y | Y  >4,000 women | Y | Y  Repeat birth by 24 months after index birth | 5  High |
| 8 Drayton 2000^40^ | Y | Y | N | Y  260 primiparous adolescent mothers | Y | Y  Over 4 years, 1994–1998 | 5  High |
| 9 Kan 2012^41^ | Y | N | N | Y  1,038 adolescents | Y | Y  Repeat pregnancy at 12 months after enrollment and “after 12 months” | 4  Moderate |
| 10 Ruch-Ross 1992^42^ | Y | N | N | Y  1,004 parenting or pregnant adolescents as subjects  790 adolescent mothers included in national youth survey as comparison group | Y | N  Repeat pregnancy by 12 months of index birth | 3  Moderate |
| 11 Feldman 2009^43^ | Y | N  Randomization of treatment and comparison groups | Y | Y  8,568 women at baseline  1,737 at final interview in 2003 | Y | Y  Repeat births during the 6 years of the Oportunidades program | 5  High |
| 12 Jones  1994^44^ | Y | N | Y | Y  210 adolescent participants, 189 comparison women 18 years or younger | Y | Y  Repeat pregnancy by 24 months  of index birth | 5  High |
| 13 Donnelly 1994^45^ | Y | N | N | Y  161 pregnant adolescents in intervention 87 pregnant adolescents served as controls | Y | Y  Repeat pregnancy at 6, 12, and 24 months after index birth | 4  Moderate |
| 14 Marsh 1991^46^ | Y | N | N | Y  335 adolescents | Y | Y  Repeat pregnancy 1 year after index birth | 4  Moderate |
| 15 Maynard 1994^47^ | Y | N | N | Y  3,400  first-time adolescent mothers | Y | Y  Repeat pregnancy after 1 and 2 years after index birth | 4  Moderate |
| **PRE-POST TEST AND OTHER NON-EXPERIMENTAL DESIGNS** | | | | | | | |
| 1 Omar 2008^48^ | Y  Retrospective review of clinic data | N | N | Y  1,386 adolescent mothers aged 11-19 | N | Y  Repeat pregnancy over 3-year program period | 3  Moderate |
| 2 Sangalang  2006^49^ | Y | N | N | Y  2,520 adolescent first-time mothers | N | Y  Repeat pregnancy at 2-year follow-up after first birth | 3  Moderate |
| 3 Schaeffer 2008^50^ | Y  Qualitative methods, focus groups | N | N | N | N | Y  Repeat pregnancy over 9-year follow-up | 2  Less Rigorous |
| 4 Carvalho Sant’Anna 2007^51^ | y | N | N | N  85 pregnant teenagers under age 18 | Y | Y  Repeat pregnancy at 23 months after index birth | 3  Moderate |
| 5 Fisher 1997^52^ | Y | N | N | Y  230 adolescents in health setting; 311 adolescents in school setting | Y | Y  Repeat pregnancy at  12 months after index birth | 4  Moderate |
| 6 Cox 2012^53^ | Y | N | N  Used benchmark comparison data from published studies | Y  I81 adolescent mothers | Y | Y  Repeat pregnancy at 12 and 24 months after index birth | 4  Moderate |
| 7 Sadler 2007^54^ | Y | N | N | N  65 adolescent mothers | Y | Y  Repeat birth at 24 months after index birth | 3  Moderate |
| 8 Brown 1999^55^ | N | N | N | N  65 adolescents under age 16 | N | Y  Repeat pregnancy during 5-year implementation period | 1  Less Rigorous |
| 9 Kuziel-Perri 1992^56^ | Y | N | N | N  52 adolescents  aged 12-19 | Y | Y  Repeat pregnancy at 2 and 4 years after index birth | 3  Moderate |
| **STUDIES MEASURING CONTRACEPTIVE CONTINUATION RATES FOR AT LEAST 2 YEARS** | | | | | | | |
| 1 Kincaid 2000^57^ | Y | N  Catchment areas randomly selected | Y | Y  1,862 women aged 14–49 | Y | Y  Continuation with contraception for  2.5 years | 5  High |

^a^ Outcomes of interest consisted of measurement of repeat pregnancy by 9, 12, 18, or 24 months after the index birth (or by 6 months after the index birth for evaluations of interventions involving the Lactational Amenorrhea Method); repeat births occurring less than 24 months after the index birth; 2-year contraceptive continuation rates; or measurement of birth or pregnancy during 3–5 years of program implementation.

^b^ High quality=5–6 “yes” assessments on the quality criteria; moderate=3–4 “yes”; less rigorous=1–2 “yes.”
