# Supplementary material for: Interventions for Preventing Unintended, Rapid Repeat Pregnancy Among Adolescents: A Review of the Evidence and Lessons From High-Quality Evaluations
Source: Glob Health Sci Pract. 2017 Dec 28;5(4):547–70. doi: 10.9745/GHSP-D-17-00131 (PMC5752603; doi:10.9745/GHSP-D-17-00131)
Supplement: Supplement Table 1 [file 17-00131-Norton-Supplement-Table2.docx]

**SUPPLEMENT TABLE 2.** Intervention Impact on Rapid Repeat Pregnancy or Birth, by Level of Impact and Quality of the Evaluation (N=40)

| **Evaluation and Country** | **Outcome Measured and Time Frame After Index Birth** | **Sample Size and Study Design** | **Intervention Description** | **Impact** |
| --- | --- | --- | --- | --- |
| **STATISTICALLY SIGNIFICANT IMPACT (N=17)** | | | | |
| **High-Quality Evaluations (n=14)** | | | | |
| Ahmed 2015^39^  Bangladesh | Birth  24 months | 4,083 women  QED | Community-based postpartum FP; strong SBC component focused on healthy spacing/fertility return before menses return targeted to woman and family; strong LAM education and transition to another modern method at 6 months | Rate of reporting a short birth interval of <24 months was significantly lower in intervention (14%) vs. control (17%) (*P*<.01). The CPR was significantly higher in the first 24 months postpartum in intervention (46%) vs. control (35%) (*P*<.001), and the odds of a short birth interval of <24 months were reduced by 23%. |
| Shaaban 2012^22^  Egypt | Pregnancy  6 months | 1,158 women  RCT | LAM and EC provision to, and training on use of EC, should intercourse occur when one of the LAM conditions is not met, for postpartum women in intervention group. LAM-only training in comparison group | I=2 pregnancies (0.8%)  C=29 pregnancies (5.0%)  *P*<.001 |
| Sullivan 1992^18^  US | Pregnancy  18 months | 243 adolescents  RCT | Health care model delivered at teen baby clinic for teen mothers including social workers, pediatrician, and referral for contraceptive service provision, focused on prevention of repeat pregnancy, return to school, immunizations, and reduced use of emergency room. | I=13/108 (12%)  C=32/113 (28%)  *P*<.003 |
| Black 2006^19^  US | Birth  24 months | 149 adolescents  RCT | Postpartum home-visitation mentoring intervention; curriculum delivered every other week until infant’s first birthday by women from community who served as mentors. Curriculum emphasized negotiation skills, adolescent development, and parenting. | I=8/70 (11%)  C=19/79 (24%)  *P*<.05  Having ≥2 intervention visits increased the odds of not having a second infant more than threefold. Only 1 mother who had 6 visits had a second birth. |
| Barnet 2009^20^  US | Birth  24 months | 235 adolescents  RCT | Computer-assisted motivational interviewing intervention (counseling style that emphasizes personal goals and self-efficacy) coupled with home visitation to prevent rapid repeat pregnancies. | Controlling for baseline group differences, the HR for repeat births was significantly lower in 1 of 2 intervention groups vs. control; HR=0.4 (*P*<.05) |
| Martin 2011^21^  UK | Pregnancy  24 months | 227 adolescents  RCT | Training for adolescents in “implementation intention formation” (if-then planning) in relation to use of contraceptives. | I=14/115 (7%)  C= 8/112 (12%)  *P*<. 02 |
| Katz 2011^23^  US | Pregnancy  24 months | 249 adolescents  RCT | Intensive cell phone counseling intervention to prevent subsequent teen pregnancies by strengthening healthy relationships and reproductive practices, positive youth assets, and teen’s own goals and needs. | Ages 15–17:  I=26%  C=39%  Participants ages 15–17 at delivery showed significant reduction in subsequent pregnancy with increased levels of intervention exposure (*P*<.01). |
| Olds 2002^24^  US | Pregnancy  24 months | 735 women with no previous live birth  RCT | Nurse home-visitation intervention to plan the next pregnancy, improve health behaviors, prevent rapid repeat pregnancies, improve parent care of children, and maternal life course development. | I=29%  C=41%  *P*<.02 |
| Drayton 2000^40^  Jamaica | Pregnancy | 260 adolescents  Historical cohort study (calculated cumulative incidence of repeat pregnancy during program follow-up period, 1995–1998) | Classroom instruction, skills training, and job placement | I=32/87 (37%)  C=104/173 (60%)  Program participation linked with significant independent effect on incidence of repeat pregnancies (*P*<.05). |
| Kincaid 2000^57^  Bangladesh | Continued use of contraception over 2.5 years | 860 women  Longitudinal cohort study | Social networks model in which clients discussed topics related to contraception, emphasizing discussion with husband and family, in the homes of village influentials; discussions led by trained government field workers. | Continuation rates over 2.5 years:  I=43.9%  C=25.5%  *P*<.001  After adjustment, the impact of the social network approach on contraceptive use was almost double that of conventional field worker visits. |
| Kitzman 1997^25^  US | Pregnancy  24 months | 1,139 women  RCT | Home visitation by nurses to improve newborn and child health and mental development, prevent injuries, and rapid repeat pregnancies. | I=36%  C=47%  *P*<.01 |
| Seitz 1993^33^  US | Birth  24 months | 102 adolescents  QED | Separate school for pregnant students integrated into the city school system in which social and medical services were provided in addition to educational classes. Counseling included helping parents plan for immediate and long-term future, and for adolescents participating >7 weeks, a requirement for postpartum checkup before exiting the school. | I=6/50 (12%)  C=19/52 (36%)  *P*<.005 |
| Rabin 1991^36^  US | Pregnancy occurring over 9 program years | 589 adolescents  QED | Comprehensive services including sexual education coupled with contraceptive education, the importance of sexual responsibility, and contraceptive availability and utilization/postpartum family planning provided by multidisciplinary team. | For all 9 years of the program:  I=9% repeat pregnancy  C=70% repeat pregnancy  Pregnancy declined significantly with each successive year of the program (*P*<.001). |
| Sebastian 2012^37^  India | Pregnancy  9 months | 959 women aged 15–24  QED | Community-based postpartum and behavior change intervention with strong emphasis on education on healthy pregnancy spacing. | I=10.5%  C=16.4%  *P*<.01 |
| **Moderate Quality (n=3)** | | | | |
| Key 2008^34^  US | Births  Followed mothers for at least 24 months | Subjects=63 adolescents  Propensity-matched comparison group=252  QED | Comprehensive services including intensive case management by school social worker, home visitation, peer education, and medical care. | Births by 30 months:  I=14%  C=26%  *P*<.05 |
| Solomon 1998^35^  US | Pregnancy  approximately 2 and 3 years after initial recruitment | 63 adolescents  QED | Family support center required to provide comprehensive services that include pregnancy testing, contraceptive services, primary health care services, educational services, and vocational services. | 2 years after recruitment:  I=3/34 (8%)  C=11/29 (37%)  *P*<.006  3 years after recruitment:  I=7/34 (20%)  C=11/29 (48%)  *P*<.02 |
| Kan 2012^41^  US | Pregnancy  within 12 months and after 12 months of program intake | 1,038 adolescents  QED | Cross-site evaluation of 12 projects required to offer 10 comprehensive health, child welfare, and contraceptive services. | Impact achieved within 12 months but not after 12 months.  Within 12 months:  I=9.8%  C=19.5%  *P*<.05  After 12 months:  I=16.4%  C=12.8% |
| **POSITIVE TRENDS OR OUTCOMES BUT NOT A STATISTICALLY SIGNIFICANT IMPACT (N=13)** | | | | |
| **High-Quality Evaluations (n=3)** | | | | |
| Koniak-Griffen 2003^26^  US | Pregnancy and birth  24 months | 101 adolescents  RCT | Home visitation using case management approach through 1 year postpartum to improve maternal health, build maternal caretaking skills, prevent rapid repeat pregnancy, and build social competence. | I=18/56 (32%)  C=21/45 (47%)  *P*>.10 |
| Ford 2002^27^  US | Pregnancy  12 months | 282 adolescents  RCT | Peer-support intervention designed to improve long- and short-term perinatal outcomes | I=13.4%  C=15.9%  Not significant |
| Lewis 2012^38^  US | Births  36 months | 144 adolescents  QED | Intensive case management to prevent 3-year subsequent births for low-income adolescent mothers. | I=9/55 (16%)  C=10/32 (31%)  Not significant |
| **Moderate Quality (n=8)** | | | | |
| Sangalang 2006^49^  US | Births  24 months | 1,260 first-time adolescent mothers  Retrospective study using North Carolina birth certificate records | Case management by social workers and services by health professionals, including goal to prevent repeat pregnancy. | Risk of 2nd birth was about 20% less in intervention group  88% of intervention mothers did not have a second birth  85% of control mothers did not have a second birth |
| Ruch-Ross 1992^42^  US | Birth  12 months | 1,794 adolescents  QED  Analysis of participant records/comparison group drawn from National Longitudinal Survey of Youth | Home visitation for 2 years after birth and peer-support model; specific goals included delaying subsequent pregnancy. | After adjustment, comparison participants were about 1.4 times more likely to experience a subsequent pregnancy at 12 months after program enrollment than intervention participants. |
| Omar 2008^48^  US | Pregnancy  12 months | 1,004 adolescent program participants compared with 790 adolescent mothers included in national survey Retrospective review of clinic data | Clinical health services and comprehensive care for teen mother and baby, including prenatal and postnatal care, mental health services, and extensive contraceptive counseling prior to start of contraceptive use and at every clinic visit. | Of 1,386 mothers, only 11 (0.79%) experienced repeat pregnancy during 3-year program period. |
| Carvalho Sant’Anna  2007^51^  Brazil | Pregnancy  23 months | 85 adolescents under age 18  Prospective analysis based on program data | Open group discussions every other week with multidisciplinary team for pregnant teens, their partners, and adolescents who had recently given birth with goal of improving contraceptive use and preventing repeat pregnancy during adolescence. | At 2 years postpartum, only 3.5% of 85 adolescent subjects had experienced a repeat pregnancy. Use of contraceptives increased from 35.4% pre-intervention to 77% post-intervention (*P*<.05). |
| Cox 2012^53^  US | Pregnancy  24 months | 144 adolescents  Prospective single-cohort demonstration project | Comprehensive medical and social services for teen mothers provided in a “medical home” including case management, promoting prevention of subsequent pregnancies, and improving life skills. | I=24%  Compared with higher 24-month pregnancy rates found in other studies |
| Sadler 2007^54^  US | Birth  Within 2 years of the index birth | Convenience sample of 65 volunteer teen mothers, aged 14–19  Descriptive study | School-based services including daily parenting classes, outreach, transportation, legal services, family counseling, liaison with health care providers, and child care. | Overall rate of subsequent childbearing in the sample of mothers was 6% (4 students) within 2 years of the index birth. |
| Kuziel-Perri  1992^56^  US | Pregnancy  4 years | 52 adolescents  4-year follow-up evaluation impact study | Eight services, including comprehensive pre- and post-birth services, including family planning methods, provided by nonprofit agency that had reputation of effective service provision. | Over 4 years, 13.5% of 52 adolescents experienced a repeat pregnancy, 57% within 24 months of first delivery. |
| Fischer 1997^52^  US | Pregnancy  12 months | School setting=311 adolescents  Health setting=230 adolescents  Analysis of program data | Social work intervention with case management delivered in school and health settings to help teen mothers complete high school, develop parenting skills, and avoid additional pregnancy. | In school setting, 9% of 311 subjects experienced repeat pregnancy, while in health setting 3% of 230 subjects experienced repeat pregnancy. |
| **Less Rigorous (n=2)** | | | | |
| Brown 1999^55^  US  Descriptive study | Pregnancy  5 years | 65 adolescents | Weekly meetings and an informal program based on needs of members with an award of $1 for each day that participants did not become pregnant. | Of 65 adolescents enrolled in the program, 10 became pregnant (15% repeat pregnancy rate). This rate was substantially lower than the 30%–35% rates reported by other programs. |
| Schaeffer 2008^50^  US  Descriptive study | Pregnancy  9 years | 276 adolescents | US school-based model, with emphasis on planning the next pregnancy. Included monthly pregnancy testing and repeated asking of question: How many children do you want to have? Model included collaboration of and multidisciplinary services provided by school, health department, and community hospital, including case management, counseling, referral, and classes in school on contraception. | Over 9 years, 20 pregnancies occurred among 276 participants, resulting in a repeat pregnancy rate of  7.2%. |
| **NO IMPACT (N=10)** | | | | |
| **High-Quality Studies (n=7)** | | | | |
| Stevens-Simon 1997^31^  US | Pregnancy  6, 12, and 24 months | 286 adolescents  RCT | Participants were randomized to 4 interventions: monetary incentive and peer-support group; peer-support group only; monetary incentive only; or no intervention. | Incidence of repeat pregnancy in 3 intervention groups at 24 months:  I (1)=34/97=35.1% (peer and incentive)  I (2)=13/23=56.5% (peer only)  I (3)=35/84=41.7% (incentive only)  C=15/44=34.1% |
| Sims 2002^32^  US | Pregnancy  24 months | 99 adolescents  RCT | Intensive family support services, including weekly home visits from paraprofessional family advocate with the goals of high school completion and limiting further childbearing; barriers to using contraceptives were removed (e.g., free medical and transport services). | I=58% repeat pregnancy  C=63% repeat pregnancy |
| Barnet 2007^28^  US | Pregnancies and births  24 months | 84 adolescents  RCT | Community-based home visitation until child’s second birthday, mentoring, case management, and skills-based interactive instruction. | I=14/31, 45% repeat pregnancy  C=12/32, 38% repeat pregnancy |
| Jones 1994^44^  US | Pregnancy  24 months | 399 adolescents  QED | Prenatal education for pregnant adolescents taught by trained volunteers. | No significant group differences were found in recorded pregnancy outcome within 2 years. |
| Cherniss 1996^30^  US | Pregnancy  24 months after entrance into program | 116 adolescents  RCT | Intensive home-based family therapy intervention to increase family support for teen to ensure medical care, transportation, better housing and improved communication, and to prevent repeat pregnancy. | Intervention did not prevent high repeat pregnancy rates. Overall rate exceeded 50% 2 years following entrance in the program. |
| El-Kamary 2004^29^  US | Pregnancy  24 months after entrance into program | 643 women  RCT | Hawaii’s Healthy Start Program: home visitation to prevent abuse, neglect, and rapid repeat pregnancy, and to promote child health and development. | There was no program impact on rapid repeat pregnancy:  I=21% repeat pregnancy  C=20% repeat pregnancy |
| Feldman 2009^43^  Mexico | Births  At 1, 2, and 3 years | Women  QED | Cash transfers to female heads of household conditional upon attendance at health education sessions, children enrolled in school, and family members obtain health checkups. | The program influenced contraceptive use but did not influence birth spacing.  Both intervention and comparison groups had similar likelihood of experiencing subsequent birth (HR=1.04, *P*<.04). |
| **Moderate Quality (n=3)** | | | | |
| Donnelly 1994^45^  US | Births  12, 24 months | 248 adolescents  QED | Case management, counseling, information and education to encourage family involvement and support of adolescent mother. | The intervention did not reduce repeat pregnancy at 12 or 24 months.  At 24 months:  I=51% repeat pregnancy  C=62% repeat pregnancy |
| Marsh 1991^46^  US | Pregnancy  3 years | 335 adolescents  Institutional cohort QED | Comprehensive service program for adolescents: health, nutrition, family planning, child care, job training, housing assistance, parenting and life skills. Program goal was to delay initial and repeat pregnancies. | “The number of repeat pregnancies in one year was completely unaffected by the program.” Over 3 years, the average number of repeat pregnancies was 18%. |
| Maynard 1994^47^  US | Pregnancy  24 months | 5297 adolescents  QED | Case management was the cornerstone of the intervention; case managers were especially trained to work with teen parents and handled 50–60 cases and helped teen parents decide on education and training opportunities. All teens required to attend a series of workshops designed specifically to help teens delay subsequent pregnancies. Case managers trained to provide family planning counseling. | “Two years after enrollment, half had become pregnant within two years after the birth of their first child.” |

Abbreviations: C, control; CPR, contraceptive prevalence rate; EC, emergency contraception; FP, family planning; HR, hazard ratio; I, intervention; LAM, Lactational Amenorrhea Method; QED, quasi-experimental design; RCT, randomized controlled trial; SBC, social and behavior change.
